# SRSF3/AMOTL1 splicing axis promotes the tumorigenesis of nasopharyngeal carcinoma through regulating the nucleus translocation of YAP1

**DOI:** 10.1038/s41419-023-06034-1

**Published:** 2023-08-09

**Authors:** Xiao-Chen Xu, Jia-Xin Jiang, Ya-Qing Zhou, Shuai He, Yang Liu, Yi-Qi Li, Pan-Pan Wei, Jin-Xin Bei, Jian Sun, Chun-Ling Luo

**Affiliations:** 1 Sun Yat-sen University Cancer Center, State Key Laboratory of Oncology in South China, Collaborative Innovation Center for Cancer Medicine, Guangdong Key Laboratory of Nasopharyngeal Carcinoma Diagnosis and Therapy, Guangzhou, 510060 P. R. China; 2 CAS Key Laboratory of Quantitative Engineering Biology, Shenzhen Institute of Synthetic Biology, Shenzhen Institutes of Advanced Technology, Chinese Academy of Sciences, Shenzhen, 518055 P. R. China; 3 Department of Experimental Research, Sun Yat-sen University Cancer Center, Guangzhou, 510060 P. R. China; 4 Department of Medical Oncology, National Cancer Centre of Singapore, Singapore, Singapore; 5 The Third Affiliated Hospital, Sun Yat-sen University, Guangzhou, 510630 P. R. China

**Keywords:** Head and neck cancer, Oncogenesis

## Abstract

Dysregulation of serine/arginine splicing factors (SRSFs) and abnormal alternative splicing (AS) have been widely implicated in various cancers but scarcely investigated in nasopharyngeal carcinoma (NPC). Here we examine the expression of 12 classical SRSFs between 87 NPC and 10 control samples, revealing a significant upregulation of SRSF3 and its association with worse prognosis in NPC. Functional assays demonstrate that SRSF3 exerts an oncogenic function in NPC progression. Transcriptome analysis reveals 1,934 SRSF3-regulated AS events in genes related to cell cycle and mRNA metabolism. Among these events, we verify the generation of a long isoform of *AMOTL1 (AMOTL1-L)* through a direct bond of the SRSF3 RRM domain with the exon 12 of *AMOTL1* to promote exon inclusion. Functional studies also reveal that AMOTL1-L promotes the proliferation and migration of NPC cells, while AMOTL1-S does not. Furthermore, overexpression of AMOTL1-L, but not -S, significantly rescues the inhibitory effects of SRSF3 knockdown. Additionally, compared with AMOTL1-S, AMOTL1-L has a localization preference in the intracellular than the cell membrane, leading to a more robust interaction with YAP1 to promote nucleus translocation. Our findings identify SRSF3/AMOTL1 as a novel alternative splicing axis with pivotal roles in NPC development, which could serve as promising prognostic biomarkers and therapeutic targets for NPC.

## Introduction

Nasopharyngeal carcinoma (NPC), originating from the nasopharyngeal epithelium, is one of the most common head and neck malignancies, which has specific geographical distributions with high prevalence in southern China, Southeast Asia, and North Africa [1]. Multiple risk factors such as Epstein-Barr virus (EBV) infection, genetic susceptibility, environmental factors, and personal lifestyle have been implicated in the development of NPC [2–4]. Recent studies have also demonstrated that transcriptional regulation of cancer-related genes contributes significantly to the initiation and progression of NPC [5–10]. However, the role of post-transcriptional regulation in NPC development is poorly investigated.

Alternative splicing (AS) is a pivotal post-transcriptional regulation process that generates various mRNA transcripts and protein products with diverse functions, of which dysregulation has been involved in the regulation of multiple cancer hallmarks, including proliferation, cell mitosis, apoptosis, invasion, and immune escape [11–14]. Generally, AS process is precisely regulated by the coordination of various splicing factors, which interact with the specific cis-elements within the pre-mRNA sequence to influence the usage of different splice sites. Serine/arginine splicing factors (SRSFs), crucial splicing factors with 12 family members, contain one or two RNA-recognition motifs (RRMs) and a serine/arginine-rich (RS) domain. They usually bind to exonic splicing enhancers or intronic elements to promote or inhibit the splicing, respectively [15]. Previous studies have demonstrated that most SRSFs were extensively dysregulated with oncogenic roles through regulating the AS of various cancer-related genes in many cancers such as lung (SRSF1 [16], SRSF5 [17]), breast (SRSF3 [18]), colon (SRSF1 [19], SRSF10 [20]), liver cancers (SRSF2 [21]), and glioma (SRSF1 [22], SRSF3 [23]). However, the specific functions and underlying mechanisms of SRSFs are still elusive in NPC.

Here we systematically profiled the expression of 12 classical SRSFs in NPC samples using transcriptome analysis and revealed SRSF3 to be highly expressed and associated with a worse prognosis in NPC. We further demonstrated that SRSF3 could promote the tumorigenesis of NPC cells via regulating the AS of *AMOTL1* to generate the oncogenic transcript *AMOTL1-L*, which interacted strongly with YAP1 to induce its translocation into the nucleus. Our findings highlight a novel SRSF3/AMOTL1-L splicing axis with an important role and prognostic potential in NPC.

## Materials and methods

### Clinical samples collection

We collected fresh NPC or control tissues from patients diagnosed with NPC or rhinitis in Sun Yat-sen University Cancer Center (SYSUCC), Guangzhou, China. All fresh tissue samples were immediately frozen in liquid nitrogen and stored at −80 °C until the extraction of protein or mRNA for western blot, transcriptome sequencing and qRT-PCR, respectively. Written informed consent was obtained from all participants, and the study was approved by the Institutional Review Board of SYSUCC.

### Cell culture and reagents

Human NPC cell lines (S26 and 5–8 F) were kindly gifted by Professor Chaonan Qian at SYSUCC (Guangzhou, China). The human embryonic kidney HEK293T cells were obtained from Cell Bank of Type Culture Collection of Chinese Academy of Sciences, Chinese Academy of Sciences. All cells were maintained in DMEM (Dulbecco’s Modified Eagle Medium, Gibco, NY, USA) with 10% fetal bovine serum (FBS, Gibco, NY, USA) and 1% Penicillin-Streptomycin (100 nM Gibco, NY, USA), in the humidified incubator with 5% CO_2_ at 37 °C. All cell lines were routinely examined to be mycoplasma-free using the mycoplasma detection kit (Vazyme, Nanjing, China).

Primary antibodies were commercially available: PARP1, Cleaved-PARP1, CCND1, C-MYC, N-Cadherin, Vimentin, γ-H2AX, α-Tubulin (9542, 5625, 2922, 9402, 14215, 5741, 9718, 2144, Cell Signaling Technology, Danvers, USA), Ki-67 (AB_393778, BD Pharmingen™, New Jersey, USA), SRSF3, HA, PMCA, EZH2 (ab125124, ab9100, ab254025, ab191250, Abcam, Cambridge, USA), AMOTL1, YAP1 (16871-1-AP, 16871-1-AP13584-1-AP, Proteintech, Chicago, USA), GAPDH (RM2002, Ray Antibody Biotech, Beijing, China), β-actin (AC004, Abclonal, Wuhan, China), Flag-tag (F1804, Sigma-Aldrich, St. Louis, USA). Secondary antibodies were HRP-linked anti-mouse IgG and anti-rabbit IgG (7076, 7074, Cell Signaling Technology, Danvers, USA).

### RNA extraction, RT-PCR and RT-qPCR

The total RNAs were extracted from NPC tissues, noncancerous tissues, or cell lines using Trizol reagent (Invitrogen, California, USA) following the manufacturer’s protocols. Then, total RNAs were reverse-transcribed into cDNA using oligo (dT) primers and M-MLV Reverse Transcriptase (Promega, Madison, WI, USA) according to the manufacturer’s procedures. RT-PCR was performed with the amplification of cDNA and the products were separated on 1% agarose gels with image capture using Bio-Rad ChemiDoc Touch (Hercules, CA, USA). Quantitative RT-PCR (RT-qPCR) was achieved with the SYBR Premix Ex Taq kit (Takara, Tokyo, Japan). The primers for amplification were listed in Table S1.

### siRNAs, plasmids construction and lentivirus packaging

siRNAs specifically against SRSF3, AMOTL1-L and YAP1 (Gene Pharma, Shanghai, China) were transfected into NPC cell lines with Lipofectamine RNAiMAX (Invitrogen, Carlsbad, CA, USA) for 48 h according to the manufacture’s procedures. Plasmids containing HA-tagged wild-type SRSF3 (SRSF3-WT) and SRSF3 domain deletion mutants (SRSF3-ΔRRM1, -ΔRRM2, -ΔRS1 and-ΔRS2) were constructed into the pcDNA3.1-HA vector. To construct the AMOTL1 splicing reporter, the fragments spanning the genome sequences from exon 11 to exon 13 were cloned into the pcDNA3.1 vector. For co-IP assay, Flag-YAP1 was cloned into the pCMV-Tag2B vector. HA-AMOTL1-L/S were constructed with pcDNA3.1 vector. shRNAs specifically targeting SRSF3 (sh-SRSF3) or exon 12 of AMOTL1 (sh- AMOTL1-L) were constructed with PLKO.1-puro plasmids. Full length cDNAs of SRSF3, AMOTL1-L or AMOTL1-S were independently cloned into the pCDH-puro lentiviral vectors. 293 T cells were transiently transfected with above plasmids according to the manufacture’s protocols and the supernatant media containing lentivirus were collected to infect NPC cell lines, following selection with puromycin (2 μg/mL) at least for one week.

### Western blotting

Tissues or cells were lysed in ice-cold cell lysis buffer (Cell Signaling Technology, Danvers, USA) containing 1x protease inhibitor (Beyotime, Shanghai, China). After centrifugation at 14,000 g for 10 min at 4 °C, total proteins were boiled in 1x SDS loading buffer at 100 °C for 10 min. Protein samples were then resolved by SDS-PAGE and transferred to polyvinylidene difluoride (PVDF) membrane (Merck Millipore, Billerica, MA, USA). After blocking with 5% bovine serum albumin (BSA; Sangon Biotech, Shanghai, China) in tris-buffered saline and Tween 20 (TBST) buffer for 1 h at the room temperature, the membranes were incubated with specific primary antibodies diluted in blocking buffer at 4 °C overnight. Next day, secondary antibodies conjugated to horseradish peroxidase (HRP) were used at room temperature for 1 h. Proteins were then detected with Fdbio-Dura ECL kit (Fdbio science, HangZhou, China) and quantified by Bio-Rad ChemiDoc Touch (Hercules, CA, USA).

### Immunohistochemistry (IHC) and evaluation

Paraffin slides of NPC tissues were dried at 60 °C in the oven for 1.5–2 h and then deparaffinized with xylene for 15 min at twice. For rehydration, the sections were immersed successively in 100%, 95%, 80%, 70% ethanol and distilled water. After boiling slides in sodium citrate solution for antigen retrieval, the slides were then treated with 0.3% hydrogen peroxide to quench the endogenous peroxidase activity, and followed by incubation with primary antibodies at 4 °C overnight. Next day, tissue sections were incubated with secondary antibodies for 1 h at room temperature, and then DAB chromogenic immunoprecipitation was performed. All sections were processing with hematoxylin for counterstaining. IHC evaluation was based on the staining intensity and percentage of stained cells, which was independently completed by two pathologists at SYSUCC.

### Immunofluorescence (IF)

IF was conducted to evaluate the protein expression and localization of candidate genes with specific antibodies as reported previously [8]. In brief, cells cultured in the glass bottom dishes were washed with phosphate-buffered saline (PBS) buffer, and then fixed in 4% paraformaldehyde for 15 min at room temperature. After permeabilizing with 0.1% Triton X-100 in PBS buffer PBST for 10 min, cells were blocked with 5% goat serum in PBST buffer for 1 h at room temperature, followed by incubation with primary antibodies at 4 °C overnight. After three washes with PBST buffer, cells were incubated with secondary antibodies diluted in the blocking buffer for 1 h at room temperature. The primary antibodies used for IF were anti-Ki-67, anti-cleaved PARP1, anti-Flag and anti-HA tag. The fluorescent secondary antibodies were goat anti-mouse IgG (Alexa Fluor 568, Invitrogen, Carlsbad, CA, USA) and goat anti-rabbit IgG (Alexa Fluor 488, Invitrogen, Carlsbad, CA, USA). Afterwards, cell nucleus was counterstained with antifade mounting medium and 4′,6-diamidino-2-phenylindole (DAPI) for 10 min and the images were then captured with a confocal laser scanning microscope (Carl Zeiss, Microscope 880, Jena, Germany).

### RNA immunoprecipitation (RIP)

RIP was used to examine the internal binding of SRSF3 and its mutants with pre-mRNA of AMOTL1 as described previously [8]. In brief, S26 cells were transiently transfected with SRSF3-HA and relative mutant plasmids or empty vectors as control. After 48 h, RIP was performed using Magna RIP kit (Merck Millipore, Billerica, MA, USA) according to the manufacturer’s instructions. Subsequently, RNA enrichment was measured by RT-PCR with specific primers targeting different exons of AMOTL1 (Table S1).

### Co-immunoprecipitation (co-IP)

For co-IP, 293 T cells were transfected with HA-AMOTL1-L/S and Flag-YAP1 plasmids using Lipofectamine 2000 according to the manufactures’ instructions. After 48 h, cells were lysed in cell lysis buffer (Cell Signaling Technology, Danvers, USA) with 1x protease inhibitor cocktail (Beyotime, Shanghai, China) for 30 min on ice. With centrifugation at 14,000 g for 10 min at 4 °C, the supernatant containing total proteins were collected and incubated with anti-Flag or anti-HA antibodies at 4 °C for 4 h. Afterwards, the protein A/G beads (Sigma-Aldrich, St. Louis, USA) pretreated with cell lysis buffer were added to the mixture at 4 °C overnight with rotation. Next day, the beads were washed with cell lysis buffer for five times, followed by boiling with 1x sodium dodecyl- sulfate (SDS) buffer for 10 min. Then, the immunocomplexes were subjected to analyze the expression of AMOTL1-L/S and YAP1 with corresponding antibodies by western blotting assay.

### Cell proliferation and apoptosis

For cell growth curves, 1×10^5^ S26 or 5–8 F cells were plated in the 12-well plates with three-time repetition and cultured for 24, 48, and 72 h. At every time point, the cells were collected and calculated by the cell counter (Cellometer Auto 1000, Nexcelom Bioscience, Boston, USA,). For colony formation assay, 3 ×10^3^ S26 or 5–8 F cells were seeded into 6-well plates in triplicate and grown at 37 °C in humidified incubator for 8-10 days. Afterwards, colonies were fixed with 4% paraformaldehyde solution for 15 min and stained with crystal violet for 15 min at room temperature, followed with visualizing by the Bio-Rad ChemiDoc Touch (Hercules, CA, USA). For cell apoptosis assay, NPC cells transfected with indicated siRNAs were detected with FITC Annexin V apoptosis detection kit I (BD Biosciences, New Jersey, USA) according to the manufacturer’s procedures, followed by flow cytometry and analysis with FlowJo (version 10.4).

### Cell migration

For transwell assay, 6 × 10^4^ NPC cells were plated into the transwell chambers (8 µm pores, Corning, NY, USA) with serum-free medium, and the chamber was placed at 24-well plates containing 600 µL DMEM suppled with 10% FBS. After 16–20 h, cells traversed the membrane were fixed in 4% paraformaldehyde for 15 min and stained with crystal violet solution for 15 min and subsequently imaged. For wound healing assay, 1 × 10^5^ S26 or 5–8 F cells were seeded in a 12-well plate with a culture-insert, which were further removed to form a defined cell-free gap. Cells were then photographed at 0, 24, and 48 h with an inverted microscope (IX73; Olympus, Tokyo, Japan).

### In vivo xenograft models

1×10^6^ S26 cells were resuspended in ice-old 1x PBS and mixed with Matrigel (0.20 v/v, Corning Incorporated, Corning, USA) and injected subcutaneously into the flanks of 6-week-old male BALB/c nude mice (Beijing Vital River Laboratory Animal Technology, Beijing, China), which grown in the Specific Pathogen Free (SPF) environment. All mice were randomly divided into several groups with 5 mice in each group. Macroscopic observation and tumor volume measurement using a caliper were performed twice a week. After 4 weeks, all mice were sacrificed, and tumor tissues were carefully dissected and weighed. Tumor volume was calculated following the formula: tumor volume (mm^3^) = length (mm) × (width (mm))^2^/2. For animal studies in vivo, all experiments were performed in strict accordance with the instructions approved by the Institutional Animal Care and Use Committee of Sun Yat-sen University.

### RNA-seq analysis

Total RNAs of S26 cells transiently transfected with siRNAs against SRSF3 or control siRNA for 48 h were isolated using the RNeasy Mini Kit (Qiagen, Duesseldorf, Germany) according to the manufacture’s protocols. Ribosomal RNAs were removed from total RNAs by using the Ribo-Zero Magnetic kit (Illumina, San Diego, USA). Subsequently, 1 μg RNA was used for library construction with TruSeq RNA Library Prep Kit (Illumina, San Diego, USA) according to the manufacturer’s instructions. The libraries were sequenced on the Hiseq X sequencer (Illumina, San Diego, USA) using pair-ends of 150 bp (GSE227503). To analyze the gene expression, Bowtie 2 [24] was used to map all reads to human reference genome (UCSC hg38 version). With removal of ribosomal RNAs, the transcript expression of each gene was quantitated using HTseq [25]. For alternative splicing analysis, CASH software was utilized to analyze the mapped reads as described previously [21]. PSI (percent spliced in) values were calculated based on the number of reads supporting inclusion or exclusion events.

### Statistical analysis

All grouped data were typically presented as mean ± standard deviation (SD), unless otherwise indicated. Statistical analyses were carried out using GraphPad Prism version 7. Data were analyzed by Student’s two-tailed *t*-test (*P < 0.05, **P < 0.01, ***P < 0.001, ****P < 0.0001). Pearson correlation analysis was used to analyze the correlation between the PSI of AMOTL1 and SRSF3 expression. Survival curves were calculated by Kaplan–Meier methods, with comparisons using the log-rank test.

## Results

### Upregulation of SRSF3 and its association with poor survival in NPC

**Fig. 1:**
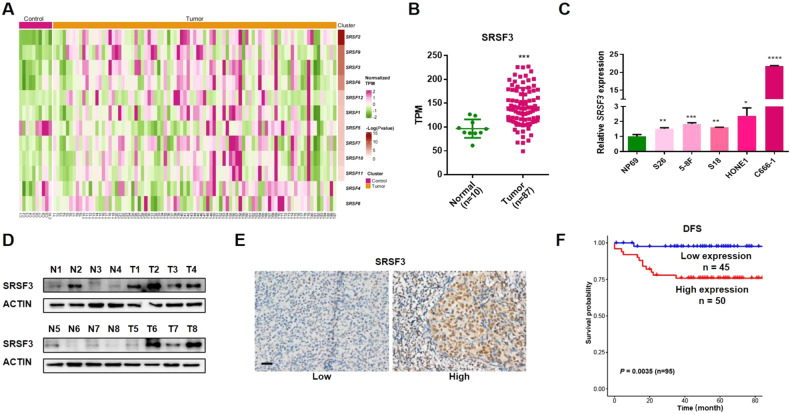
SRSF3 is highly expressed and associated with worse prognosis in NPC. **A** Heatmap results showed the transcriptome expression levels of 12 classical SRSFs in NPC tissues (*n* = 87) and control tissues (*n* = 10). **B** RNA-seq data presented as TPM described in **A** showed the expression of *SRSF3* in tumor and control tissues. **C** qRT-PCR was performed to detect the mRNA expression of *SRSF3* in NPC cell lines compared with immortalized non-cancerous nasopharyngeal epithelial cells (NP69). **D** Western blotting assay showed the protein levels of SRSF3 in another NPC cohort. ACTIN was used as an internal control. **E** IHC staining was performed to evaluate the protein expression of SRSF3 in NPC samples (*n* = 95). **F** Kaplan–Meier survival analysis showed the correlation between the protein expression of SRSF3 and disease-free survival of NPC patients described in **F**. Scale bar, 200 μm. *P < 0.05, **P < 0.01, ***P < 0.001.

To systematically explore the roles of SRSFs in NPC, we first analyzed the mRNA expression levels of 12 classical SRSFs in 87 NPC tumors and 10 non-cancerous control samples using in-house transcriptome data, among which *SRSF2*, *SRSF3*, and *SRSF9* were found to be upregulated in NPC samples compared with control samples (*P* < 0.01; Fig. 1A, B, and Supplementary Fig. 1A). Subsequently, we performed real-time quantitative PCR (RT-qPCR) assay and observed that only *SRSF3* was highly expressed in NPC cell lines compared with a normal nasopharyngeal epithelium cell line (NP69; Fig. 1C and Supplementary Fig. 1B). Furthermore, western blotting assays confirmed the remarkably high expression of SRSF3 in NPC tumor tissues compared with control tissues (Fig. 1D, Supplementary File 1). To assess the clinical significance of SRSF3 expression, we examined the protein expression of SRSF3 in NPC using immunohistochemistry (IHC) assays and performed survival analysis, which revealed that higher expression of SRSF3 was significantly associated with worse prognosis in individuals diagnosed with NPC (Fig. 1E, F, Supplementary Fig. 1C). Moreover, upregulated SRSF3 expression was remarkably correlated with metastasis and tumor grade of the patient with NPC (Table 1). Taken together, these observations suggest SRSF3 as a potential oncogene contributing to NPC development.

**Table 1:** Clinical characteristics of NPC patients.

Groups	Low expression	High expression	Test of significance
	(*n* = 45)	(*n* = 50)	
Gender			
Male	34 (75.6%)	40 (80%)	
Female	11 (24.4%)	10 (20%)	*P* = 0.7843
Age			
<50	25 (55.6%)	30 (60%)	
≥50	20 (44.4%)	20 (40%)	*P* = 0.8181
N stage			
N0	5 (11.1%)	4 (8%)	
Nx	40 (88.9%)	46 (92%)	*P* = 0.868
Distant Metastasis			
0	44 (97.8%)	41 (82%)	
1	1 (2.2%)	9 (18%)	*P* = 0.0302*
Stage			
Early (1–2)	8 (17.8%)	5 (10%)	
Advanced (3-4)	37 (82.2%)	45 (90%)	*P* = 0.4223
T Stage			
1	7 (15.6%)	0 (0%)	
2	6 (13.3%)	12 (24%)	
3	20 (44.4%)	24 (48%)	
4	12 (26.7%)	14 (28%)	*P* = 0.0258*

Statistical analysis showed the relationship between the protein expression of SRSF3 and the clinicopathological characteristics of NPC patients (*n* = 95). *P < 0.05.

### Tumorigenic role of SRSF3 in NPC progression

**Fig. 2:**
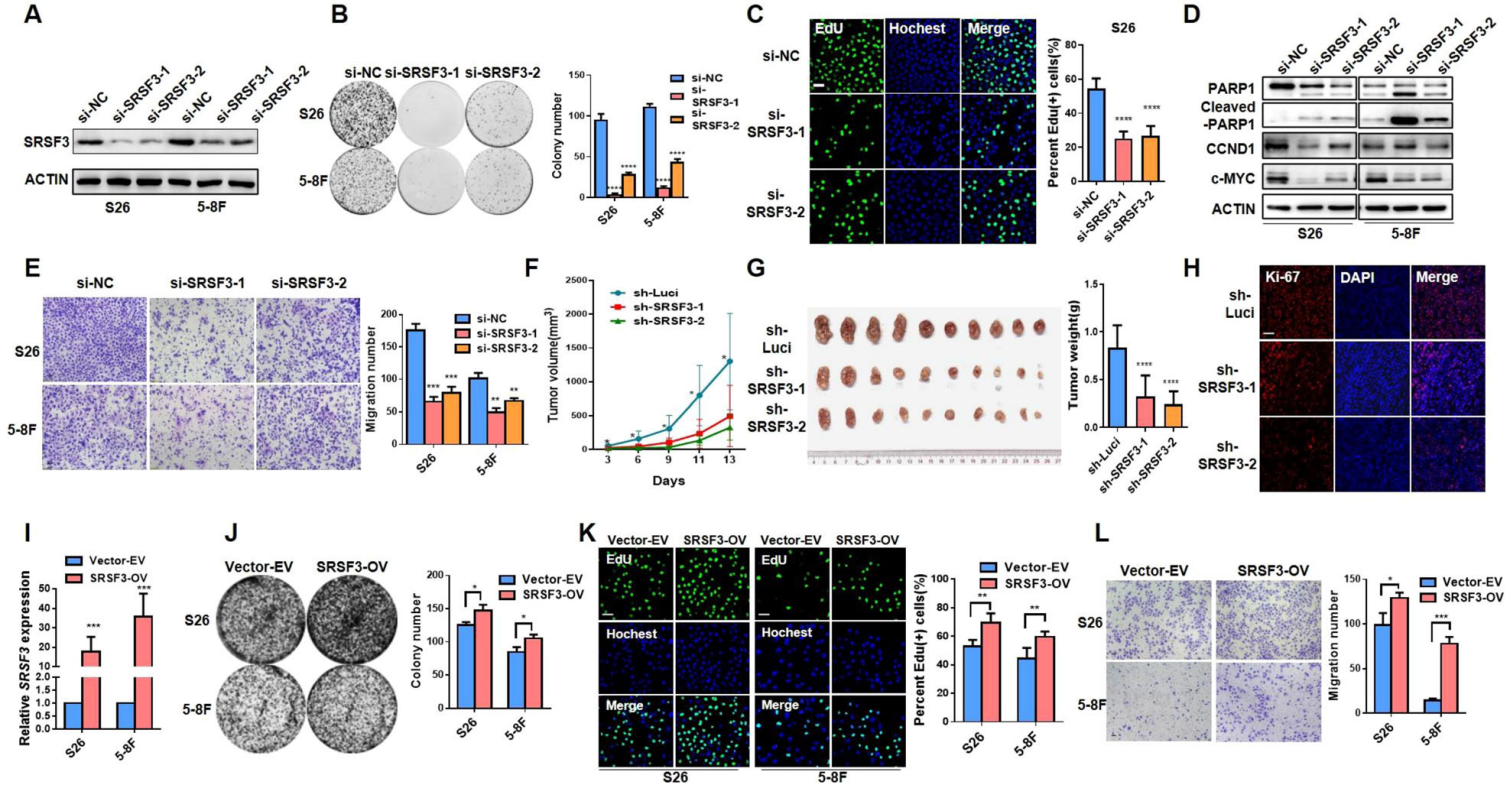
SRSF3 acts as an oncogenic role in NPC cells. **A** S26 and 5–8 F cells were transiently transfected with SRSF3 siRNAs (si-SRSF31 and si-SRSF3-2) and control siRNA (si-NC). Western blotting was performed to evaluated the knockdown efficiency of SRSF3, with ACTIN as control. **B** Colony formation assay was performed with cells described in **A**, and the statistical analysis was shown at the right. **C** Representative images of EdU staining for S26 cells described in A (left) and the corresponding statistical analysis was exhibited at the right. **D** Western blotting assay demonstrated the protein levels of PARP1, cleaved-PARP1, CCND1, and C-MYC in S26 and 5–8 F cells described in **A**. ACTIN was used as control. **E** Representative images of transwell assay in NPC cells described in **A**. Corresponding statistical analysis was presented at the right. **F** The growth curves of xenograft tumors derived from S26 cells stably expressing SRSF3 shRNAs (sh-SRSF3-1 and sh-SRSF3-2) or control shRNA (sh-Luci) lentiviruses. **G** Tumor size (left) and weight (right) for the xenograft tumors excised from **F** were presented. **H** Immunofluorescence staining of Ki-67 in paraffin-embedded xenograft tumors presented in **F**. **I** S26 and 5–8 F cells were infected with lentivirus expressing SRSF3 or empty vector. The overexpression level of SRSF was confirmed by qRT-PCR assay. **J** Colony formation assay was performed with cells described in **I** and the statistical analysis was shown at the right. **K** EdU staining was performed with the cells described in **I** and the percentage of EdU (+) cells in each group was presented at the right panel. **L** Transwell assay was performed to evaluated the migration ability of S26 and 5–8 F cells described in I with statistical analysis showing at the right. Scale bar, 100 μm. *P < 0.05, **P < 0.01, ***P < 0.001.

To further investigate the biological function of SRSF3 in NPC, we knocked down the expression of SRSF3 using siRNAs in S26 and 5–8 F cells (Fig. 2A, Supplementary File 1). Colony formation (Fig. 2B) and cell growth curve assays (Supplementary Fig. 2A) demonstrated that knockdown of SRSF3 significantly decreased the proliferation ability of NPC cells, which was further confirmed by obviously reduced numbers of SRSF3-knockdown cells stained with EdU, a well-known proliferation marker (Fig. 2C). Meanwhile, western blotting assay revealed that the expression of CCND1 and c-MYC, proliferation markers, was decreased in the SRSF3-knockdown cells (Fig. 2D, Supplementary File 1). Furthermore, SRSF3 knockdown markedly induced apoptosis of NPC cells as revealed by the increased Annexin V/propidium iodide (PI) staining (Supplementary Fig. 2B) and proteolytic cleavage of PARP activation (Fig. 2D, Supplementary File 1). Moreover, transwell and wound healing assays revealed that SRSF3 knockdown inhibited the migration of NPC cells (Fig. 2E, Supplementary Fig. 2C), further confirmed by reduced expression of N-cadherin and Vimentin, well-known migration markers (Supplementary Fig. 2D, Supplementary File 1). These observations suggest an oncogenic role of SRSF3 in NPC.

To explore the tumorigenic function of SRSF3 in vivo, we established xenograft models with S26 cells stably expressing shRNAs targeting SRSF3 (sh-SRSF3-1 and -2) or control shRNA (sh-Luci). We observed remarkable reductions in tumor volume and weight in SRSF3-knockdown groups compared with the control groups (Fig. 2F, G). Consistently, the proportion of Ki-67(+) cells, indicating high proliferative activity, was significantly decreased in the SRSF3-knockdown groups (Fig. 2H). Moreover, to further validate the oncogenic role of SRSF3 in NPC, we constructed NPC cell lines with stable overexpression of SRSF3 (Fig. 2I and Supplementary Fig. 2E, Supplementary File 1) and we observed remarkably enhanced proliferation and migration capacities of the NPC cells (Fig. 2J–L). Taken together, these findings strongly suggest that SRSF3 is essential for the proliferation, migration, and tumorigenesis of NPC cells.

### Global landscape of AS events regulated by SRSF3 in NPC cells

**Fig. 3:**
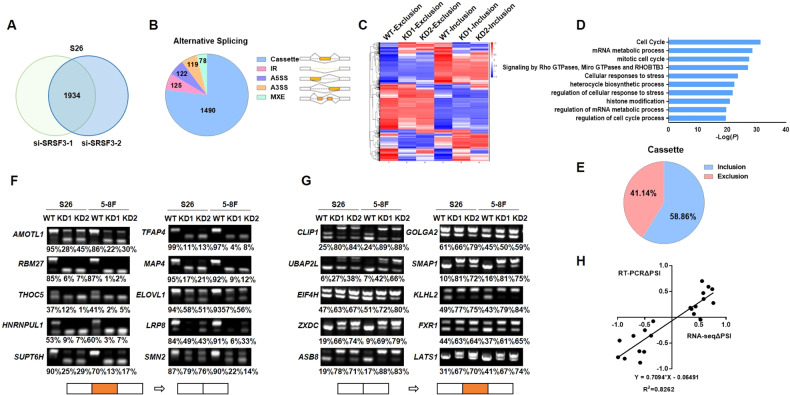
Global landscape of SRSF3-regulated AS events in NPC cells. **A** RNA-seq was performed with S26 cells transiently transfected with siRNAs against SRSF3 or control siRNA to analyze the differential AS events influenced by SRSF3 knockdown. Venn diagram showed the total number of AS events altered in both two SRSF3 siRNAs, compared with control group. **B** Quantification of AS events described in **A**, which were classified into five categories: Cassette, retained intron (IR), alternative 5′-splice site (A5SS), alternative 3′- splice site (A3SS), and mutually exclusive exon (MXE). **C** Heatmap showed the global alteration of SRSF3-affected AS events demonstrated by the PSI. **D** Gene ontology pathways analysis of SRSF3-regulated splicing events. **E** The cassette splicing events described in **B** were classified into exon inclusion and exclusion. **F**, **G** RT-PCR was performed to validate the SRSF3-regulated AS events described in **E**. Representative graphs illustrated the exon exclusion (**F**) and exon inclusion (**G**) with NPC cells transfected with SRSF3 siRNAs. The PSI of each gene was presented on the corresponding bottom lane. The boxes on the bottom represents the exons, of which red boxes indicate alternative exons. **H** The correlation between the ΔPSI of the RNA-seq analysis and RT-PCR validation presented in **F**, **G** was shown.

Considering that SRSF3 is a classical splicing factor [26], we investigated how SRSF3 executes its tumorigenic function through alternative splicing regulation. Transcriptome analysis revealed a total of 1,934 AS events affected by SRSF3 knockdown in S26 cells (KD1 and KD2 groups; Fig. 3A), including 1,490 cassette exons, 125 retained introns (IR), 122 alternative 5’-splice sites (A5SS), 119 alternative 3’-splice sites (A3SS), and 78 mutually exclusive exons (MXE, Fig. 3B). Heatmap analysis revealed that most AS events changed significantly either in inclusion or exclusion of alternative exons (Fig. 3C). Moreover, Gene Ontology (GO) analysis revealed that the SRSF3-affected AS events were functionally enriched in signaling pathways including cell cycle and regulation of mRNA metabolic process (Fig. 3D).

Among the AS events of cassette exons, the proportions of exon inclusion (58.86% or 877/1,490) and exclusion (41.14% or 613/1,490) were comparative (Fig. 3E), suggesting a dual role of SRSF3 as both the splicing activator and repressor. Subsequently, we selected 20 top AS events from both groups for further validation using reverse transcription PCR (RT-PCR, see Materials and Methods for detail). We confirmed that SRSF3 either activated (Fig. 3F) or repressed (Fig. 3G) the splicing of target exons, which alterations presented by $\Delta$PSI (present spliced in) were significantly correlated with that from the transcriptome analysis (Fig. 3H). These findings strongly suggest SRSF3 as a crucial splicing factor regulating multiple AS events in NPC.

### Mutual binding sites between SRSF3 and AMOTL1 are responsible for the exon inclusion

**Fig. 4:**
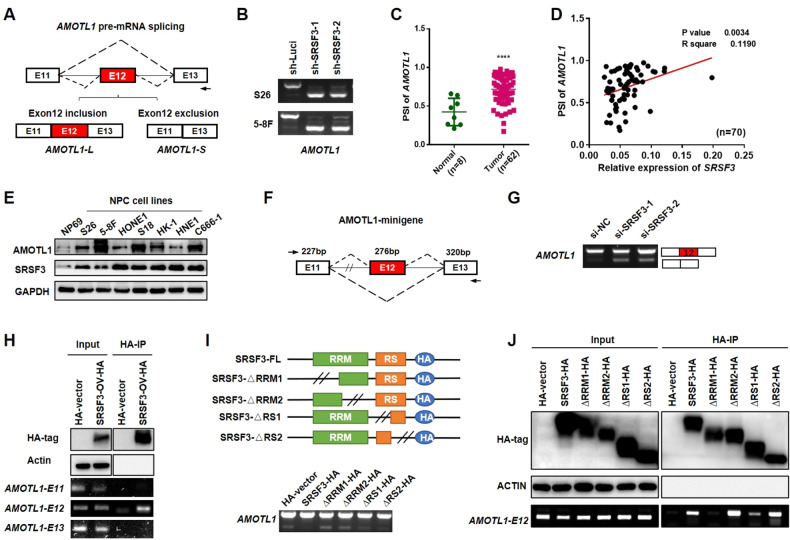
SRSF3 binds to the exon 12 of *AMOTL1* to promote its inclusion via RRM domain. **A** Schematic diagram presented the *AMOTL1* pre-mRNA splicing. The red and white boxes indicated alternative and constitutive exons, respectively. **B** RT-PCR showed the *AMOTL1* splicing in S26 and 5–8 F cells stably expressing SRSF3 shRNAs or control shRNA. **C** PSI of *AMOTL1* exon 12 in NPC samples (*n* = 62) compared with control samples (*n* = 8). **D** Pearson correlation analysis between the PSI of *AMOTL1* exon 12 and mRNA expression of SRSF3 in the samples described in **A**. **E** Western blotting results showed the protein levels of AMOTL1 and SRSF3 in NPC cell lines compared with NP69. GAPDH was used as a loading control. **F** Schematic diagram presented the AMOTL1 minigene system. **G** RT-PCR results demonstrated the splicing pattern of *AMOTL1* exon 12 in S26 cells co-transfected with SRSF3 siRNAs and AMOTL1 minigene plasmids. **H** S26 cells infected with lentivirus stably expressing SRSF3-HA or control vectors were collected for RNA immunoprecipitation (RIP) assay, which overexpression was validated by the western blotting assay. RIP-PCR was then performed to evaluate the internal binding with specific primers. **I** Schematic diagram (upper) indicated the HA-SRSF3-FL and its mutants for ΔRRM (deleting of RRM domain) and ΔRS (deleting of RS domain). S26 cells were co-transfected with siRNA targeting SRSF3 and the plasmids described above. RT-PCR was performed to detect the splicing of *AMOTL1* exon 12. **J** RIP-PCR was performed with S26 cells transfected with plasmids described in **I**. Western blotting results showed the overexpression of SRSF3 and its mutants. *P < 0.05, **P < 0.01, ***P < 0.001, ****P < 0.0001.

Among the SRSF3-affected AS events, we identified that the expression of *AMOTL1* containing exon 12 was suppressed with SRSF3 knockdown, generating from the long isoform (*AMOTL1-L*) in the control cells to the short isoform (*AMOTL1-S*; Fig. 4A, B). Furthermore, *AMOTL1-L* was upregulated in NPC samples compared with control samples (Fig. 4C), and the ratio of *AMOTL1-L/S* was positively correlated with the expression of *SRSF3* in the NPC samples (*P* = 0.034, Fig. 4D) and NPC cell lines (Fig. 4E, Supplementary File 1) compared with their corresponding controls, suggesting the regulation of SRSF3 on *AMOTL1* splicing.

To elucidate the mechanism of how SRSF3 regulates the alternative splicing of *AMOTL1* exon 12, we constructed a minigene reporter plasmid containing the genomic DNA fragment from the exon-11 to exon-13 of *AMOTL1* (Fig. 4F), followed by transient transfection into S26 cells with or without SRSF3 knockdown. We observed a significant increase of the *AMOTL-S* variant without the exon 12 fragment in the SRSF3 knockdown cells, compared with the presence of the predominant *AMOTL1-L* variant in the WT cells, suggesting that the inclusion of exon 12 of *AMOTL1* is SRSF3 dependent (Fig. 4G). Next, RNA immunoprecipitation (RIP) and RT-PCR revealed that SRSF3 strongly bonded to the alternative exons 12 but not 11 and 13 of *AMOTL1* (Fig. 4H, Supplementary File 1). These observations suggest that SRSF3 binds directly to the exon 12 of *AMOLT1* to promote its inclusion.

To further explore the regulatory mechanisms, we examined the binding sites of SRSF3 to *AMOTL1*. We transiently overexpressed wild-type (SRSF3-WT) or mutants at any of the two functional domains of SRSF3 (SRSF3-ΔRRM1, -ΔRRM2 or -ΔRS1, -ΔRS2) in S26 cells in combinations of *AMOTL1* minigenes to examine their regulation on *AMOTL1* splicing (Fig. 4I, upper). RT-PCR assay revealed that deletion of the functional RRM domains in SRSF3 (SRSF3-ΔRRM1 and-ΔRRM2) inhibited its promotion on the exon 12 inclusion compared with SRSF3-WT and SRSF3-ΔRS mutants (Fig. 4I, bottom). Consistent with this, RIP-PCR assay also demonstrated that SRSF3 bonded to the exon 12 of *AMOTL1* through the RRM domain (Fig. 4J, Supplementary File 1). Taken together, these observations strongly suggest that SRSF3 binds to the alternative exon of *AMOTL1* via the RRM domain to promote its exon inclusion.

### Oncogenic function of AMOTL1-L in NPC

**Fig. 5:**
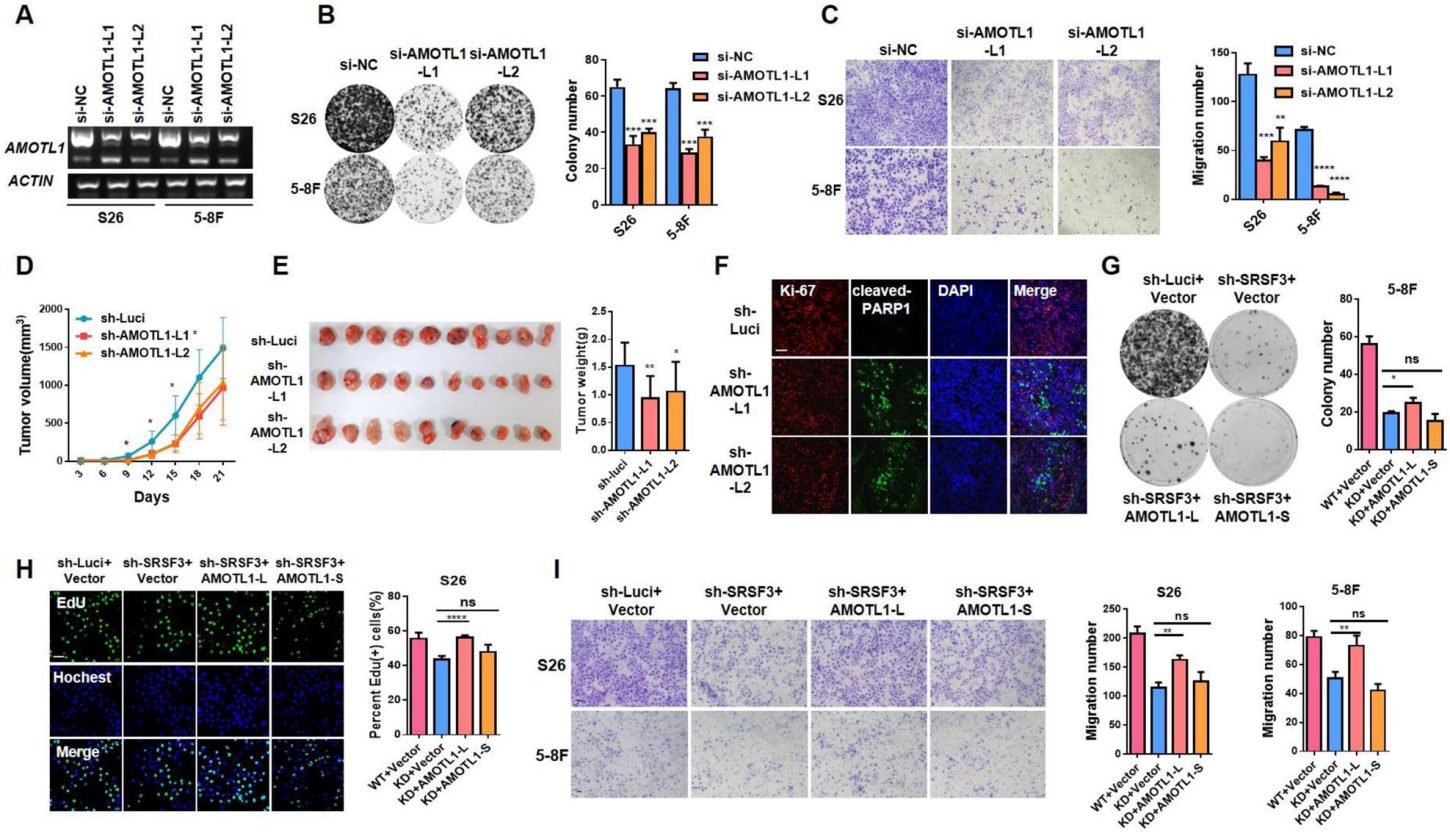
AMOTL1-L mediates the tumorigenic function of SRSF3 in NPC. **A** S26 and 5–8 F cells were transfected with siRNAs specific targeting the exon 12 of AMOTL1. RT-PCR assay was performed to detect the knockdown efficiency of AMOTL1-L, with *ACTIN* was used as control. **B** Colony formation assay was performed with cells described in **A** and the statistical analysis was shown at the right. **C** Transwell assay was performed with cells described in **A** with corresponding statistical analysis presented at the right. **D** S26 cells infected with AMOLT1-L shRNAs or control shRNA lentivirus were subcutaneously injected into the flanks of nude mice. The growth curves of the xenograft tumors were shown. **E** Tumor size (left) and weight (right) for the xenograft tumors excised from **D**. **F** Representative images of immunofluorescence staining of Ki-67 and cleaved-PARP1 in paraffin-embedded xenograft tumors shown in **E**. **G** 5–8 F cells stably expressing SRSF3 shRNAs or control shRNA were infected with lentivirus expressing AMOTL1-L/S. Colony formation assay was performed the quantification of colony numbers was shown at the right. **H** EdU staining was performed with cells described in **G** and the corresponding statistical analysis was presented at the right panel. **I** Transwell experiment was performed with cells described in **G** and the statistical graph was shown at the right. Scale bar, 100 μm. *P < 0.05, **P < 0.01, ***P < 0.001.

Given that the oncogenic SRSF3 facilitated the expression of AMOTL1-L, we next investigated whether AMOTL1-L contributes to NPC development. We first knocked down the expression of AMOTL1-L in NPC cell lines (S26 and 5–8 F) using two siRNAs specifically targeting the exon 12 of AMOTL1 (Fig. 5A and Supplementary Fig 3A, Supplementary File 1) and observed significantly inhibited proliferation ability of NPC cells with AMOTL1-L knockdown compared with the control group (Fig. 5B, Supplementary Fig. 3B), which was further confirmed by the reduced EdU (+) cells and decreased expression of CCND1 and c-MYC in the AMOTL1-L knockdown cells (Supplementary Fig. 3C, D, Supplementary File 1). Flow cytometry revealed that AMOTL1-L knockdown markedly induced apoptosis in NPC cells (Supplementary Fig. 3E). Transwell and wound healing assays demonstrated that the migration ability was obviously inhibited with AMOTL1-L depletion in NPC cells (Fig. 5C, and Supplementary Fig. 3F). Moreover, to further explore the function of AMOTL1-L in vivo, we established xenograft mouse models with subcutaneous injection of S26 cells infected with lentivirus expressing shRNAs specifically targeting the exon 12 of AMOTL1 (sh-AMOTL1-L1 and sh- AMOTL1-L2) or control shRNA (sh-Luci). We observed significantly decreased tumor volume and weight in the AMOTL1-L knockdown group compared with the control group (Fig. 5D, E), indicating that depletion of AMOTL1-L inhibited the tumor growth of NPC cells, which was further validated by the reduced Ki-67 (+) cells and induced expression of cleaved-PARP1 in the AMOTL1-L knockdown tumors. (Fig. 5F, and Supplementary Fig. 3G).

We further established cell lines (S26 and 5–8 F) with stable overexpression of AMOTL1-L, AMOTL1 -S, or empty vector (Supplementary Fig. 4A, Supplementary File 1). Colony formation assay and staining assay of proliferation marker EdU(+) revealed that overexpression of AMOTL1-L could significantly promote the cell proliferation of NPC cells, while AMOTL1-S did not (Supplementary Fig. 4B, C). Transwell assay showed that AMOTL1-L but not -S improved the migration of NPC cells (Supplementary Fig. 4D). Furthermore, Kaplan–Meier survival analysis showed that higher expression of AMOTL1-L was associated with worse prognosis of NPC patients (Supplementary Fig. 4E). Taken together, these findings strongly suggest that AMOTL1-L is essential to the proliferation, migration, and tumorigenesis of NPC cells, consistent with the function of its modulator, SRSF3.

### AMOTL1-L is responsible for the tumorigenic function of SRSF3 in NPC

Given that SRSF3 shared consistent functions with its AS product of AMOTL1-L, we probed whether the latter mediates the oncogenic function of SRSF3 in NPC. First, we established NPC cell lines with the combinations of SRSF3-knockdown and AMOTL1-L or -S (Supplementary Fig. 4E, Supplementary File 1). Colony formation and EdU (+) staining assays revealed that SRSF3-knockdown significantly decreased the proliferation of NPC cells, which was partially rescued by the overexpression of AMOTL1-L, but not AMOTL1-S (Fig. 5G, H). Transwell assay also confirmed that AMOTL1-L overexpression rescued the migration inhibitory effects caused by SRSF3 knockdown, while AMOTL1-S did not (Fig. 5I). Collectively, these observations strongly suggest that AMOTL1-L partially mediates the tumorigenic function of SRSF3 in NPC.

### Cytoplasm localization of AMOTL1-L mediates the nuclear entry of YAP1

**Fig. 6:**
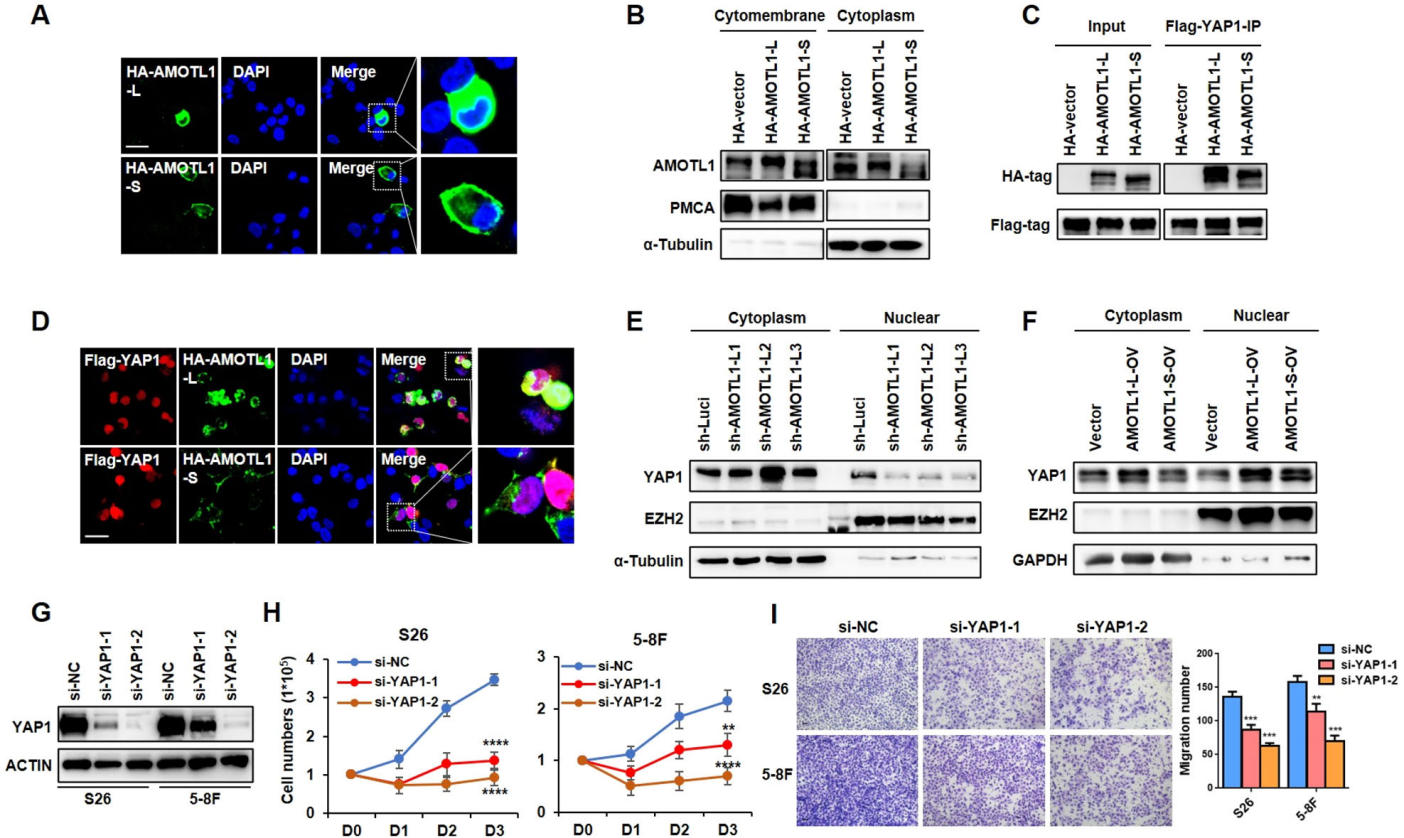
AMOTL1-L interacts with YAP1 to promote its nucleus translocation. **A** Immunofluorescence assay was performed to detect the localization of HA-tag AMOTL1-L/S in 5–8 F cells transiently transfected with HA-AMOTL1-L or HA-AMOTL1-S plasmids. **B** Western blotting analysis showed the protein level of AMOTL1 in cytomembrane and cytoplasm with 5–8 F cells described in **A**. PCMA and α-Tubulin were used as markers for cell membrane and cytoplasmic, respectively. **C** co-IP assay was performed with 293 T cells co-transfected with HA- AMOTL1-L/S and Flag-YAP1 plasmids. **D** Representative images of immunofluorescent staining of Flag-YAP1 (red) and HA-AMOTL1-L/S (green) in cells described in **C**. **E**, **F** Western blotting results showed the YAP1 expression in the cytoplasmic and nuclear of S26 cells stably expressing AMOTL1-L shRNAs (**E**) or AMOTL1-L/S overexpression lentivirus (**F**). EZH2 and α-Tubulin were used as protein markers for nuclear and cytoplasmic, respectively. **G** S26 and 5–8 F cells were transfected with YAP1 siRNAs (si-YAP1-1 and si-YAP1-2) and control siRNA (si-NC). Western blotting assay demonstrated the knockdown efficiency of YAP1 in above cells. **H** Growth curves were drawn with cells described in **G**. **I** Transwell assay was performed with cells described in **G** and the corresponding statistical analysis was presented at the right. Scale bar, 100 μm. *P < 0.05, **P < 0.01, ***P < 0.001.

We next explored the regulatory mechanisms of AMOTL1 variants with distinctive roles in NPC development. First, immunofluorescence assay revealed that AMOTL1-L and -S was preferably localized in the cytoplasm and the cell membrane of NPC cells, respectively (Fig. 6A), which was further validated by immunoblotting assays (Fig. 6B, Supplementary File 1), suggesting that such distinct cellular localizations may influence their interactions with downstream molecules and lead to distinctive functions. Considering previous findings that AMOTL1 could bind to YAP1 and promote its entry to the nucleus [27], we examined the binding ability of AMOTL1-L/S with YAP1. Immunofluorescence assay demonstrated that AMOTL1-L was strongly co-localized with YAP1 in NPC cells, while AMOTL1-S did not (Fig. 6D). Co-immunoprecipitation (co-IP) assay revealed that AMOTL1-L directly bonded with YAP1, with an obviously stronger binding than AMOTL1-S (Fig. 6C, Supplementary File 1). Nucleoplasmic separation assays revealed that knockdown or overexpression of AMOTL1-L inhibited or promoted the nuclear localization of YAP1, respectively (Fig. 6E, F, Supplementary File 1).

Next, we further explored the tumorigenic potential of YAP1 in NPC cells by knocking down the expression of YAP1 using siRNA (Fig. 6G, Supplementary File 1). Cell growth, colony formation, and transwell assays revealed that depletion of YAP1 significantly decreased the proliferation and migration of NPC cells (Fig. 6H, I, Supplementary Fig. 5), consistent with the oncogenic role of AMOTL1-L. Taken together, these observations strongly suggest that AMOTL1-L binds to and promotes nuclear entry of YAP1 to promote the tumorigenesis of NPC.

## Discussion

SRSFs have been reported to be dysregulated in various cancers as classical splicing factors, but the underlying mechanisms are still largely elusive, especially in NPC. Here, to the best of our knowledge, we performed a comprehensive investigation of SRSFs in NPC for the first time and revealed that SRSF3 acted as an oncogene in NPC. Our study revealed that SRSF3 was upregulated in NPC, with high expression associated with worse prognosis. Functional assays further revealed that SRSF3 significantly promoted the proliferation, migration, and tumorigenesis of NPC cells. Consistently, previous studies have identified SRSF3 as a proto-oncogene with high expression in various cancers [23, 28]. SRSF3 regulates multiple cancer-related genes, including *EP300*, *DDX5*, and *MAP4K4*, to increase the proliferation of sarcoma cells [29]. In glioblastoma, SRSF3 promotes the exon 7 inclusion of *ETV1* and mutually exclusive of the exon 9 of *NDE1* to promote tumorigenesis [23]. However, it has been reported that specific depletion of SRSF3 in hepatocytes leads to hepatocellular carcinoma in mice [30]. Furthermore, SRSF3 knockdown inhibits the exon 2 inclusion of CD19, thereby attenuating the effect of CAR-T therapy against this epitope and leading to high recurrence rates in B-ALL [31]. These findings indicate the double-edged functions of SRSF3 in cancer development, which may be attributed to the tissue-specific properties of SRSF3-regulated AS events with distinct functional consequences among different cancer types.

Our study revealed 1934 SRSF3-regulated AS events, with a preference for cassette exons, among which *AMOTL1* was a functional target of SRSF3. Recent studies demonstrate that AMOTL1, a member of the Angiomotin family, holds oncogenic potentials in glioma, breast, and gastric cancers [27, 32, 33]. However, it is unclear which AMOTL1 isoform contributes to the tumorigenic function, although multiple transcriptional isoforms have been identified previously [34]. We here discovered a novel *AMOTL1-L* splicing variant regulated by SRSF3 through binding to and including its alternative exon 12, supporting a previous finding that the binding motif of SRSF3 is enriched in the alternative exon [23, 26]. Moreover, mutational assay revealed that SRSF3 bonded to the target RNA through its RRM domain, consistent with the conserved function of the RRM domain in SRSFs [35, 36], further verifying its direct regulation on *AMOTL1* splicing. Additionally, we observed that the depletion of the RS domain in SRSF3 also resulted in a partial decrease in its binding capability to RNA, which may explain why the phosphorylation of the RS domain may influence the RRM exposure, consequently affecting its ability to recognize and bind to RNA [37]. Furthermore, functional results demonstrated that including the exon 12 or not in AMOTL1 (AMOTL1-L or -S, respectively) exhibited different functions in NPC. AMOTL1-L promoted the proliferation, migration, and tumorigenesis of NPC cells, nor did AMOTL1-S. Rescue assays also demonstrated that overexpression of AMOTL1-L partially rescued the inhibiting effects induced by SRSF3 knockdown, while AMOTL1-S did not. These findings strongly suggest a novel SRSF3/AMOTL1 splicing axis with distinct tumorigenic roles depending on the alternative splicing regulation in NPC development.

We further revealed that AMOTL1-L might hold its oncogenic function through the preferential cytoplasm localization and interaction with YAP1 to induce nuclear translocation. AMOTL1 is a component of tight junctions, interacting with actin to regulate cellular polarity and adhesion [38, 39]. Recent studies have established a strong link between AMOTL1 and Hippo signaling pathway [40, 41]. Specifically, AMOTL1 promotes tumorigenesis via interacting with YAP1, a vital factor of the Hippo pathway, to induce its nuclear accumulation in gastric cancer and glioma [27, 32]. As a classical transcription activator, YAP1 has been reported to act as an oncogene with a tumorigenic role in developing various cancers [42, 43]. Generally, YAP1 translocates into the nucleus to regulate downstream gene expression, and its accumulation in cytoplasm leads to degradation via ubiquitin-mediated proteolysis [44]. We observed distinct localizations of AMOTL1-L and -S in NPC cells, of which AMOTL1-L is preferably localized in the intracellular, whereas AMOTL1-S is localized in the cell membrane. Furthermore, we revealed that AMOTL1-L had a more robust binding capacity with YAP1 to promote nuclear translocation than AMOTL1-S. These findings suggest that the distinct functions of two AMOTL1 variants might be mediated through differential interacting capabilities with YAP1 to induce its nucleus entry and thus downstream functional signaling pathways.

Our study also has several limitations. First, given that the tumorigenic effect of SRSF3 could be partially rescued by AMOTL1-L, other candidate AS events regulated by SRSF3 may also play important roles in NPC development, which await further investigations. Second, the mechanism underlying the influence of the exon 12 and the precise sequence of AMOTL1 on its cytoplasmic localization remains elusive. This could be further explored by using structural prediction and experimental validations. Lastly, besides SRSF3, our transcriptomic data also revealed the upregulation of other SRSF members (SRSF2 and SRSF9) in NPC. Further study on the potential roles of SRSF2 and SRSF9 would shed light on the SRSF family in NPC development.

In summary, our study identifies a novel SRSF3/AMOTL1 splicing axis contributing to the development of NPC. Mechanistically, SRSF3 mediates the alternative splicing of AMOTL1 to generate the oncogenic transcript AMOTL1-L, which is preferably localized in the intracellular compartment and has a robust interaction with YAP1 to promote its nucleus entry in NPC, thereby exhibiting tumorigenic potentials in NPC. Our findings shed light on the potential of SRSF3/AMOTL1 as biomarkers for patient stratification and therapeutic targets in NPC.

### Reporting summary

Further information on research design is available in the Nature Research Reporting Summary linked to this article.

## Supplementary information

Supplementary Figures

Table S1

Original Data File

Confirmation of changes in Authorship

Reporting Summary

## Data Availability

All relevant data are available from the authors upon request. Key data were deposited in the Research Data Deposit public platform (RDD:RDDB2023671034; http://www.researchdata.org.cn).
