# Supplementary material for: SRSF3/AMOTL1 splicing axis promotes the tumorigenesis of nasopharyngeal carcinoma through regulating the nucleus translocation of YAP1
Source: Cell Death Dis. 2023 Aug 9;14(8):511. doi: 10.1038/s41419-023-06034-1 (PMC10412622; doi:10.1038/s41419-023-06034-1)
Supplement: Supplementary file 1 — Supplementary Figures [file 41419_2023_6034_MOESM1_ESM.docx]

**Figure S1**


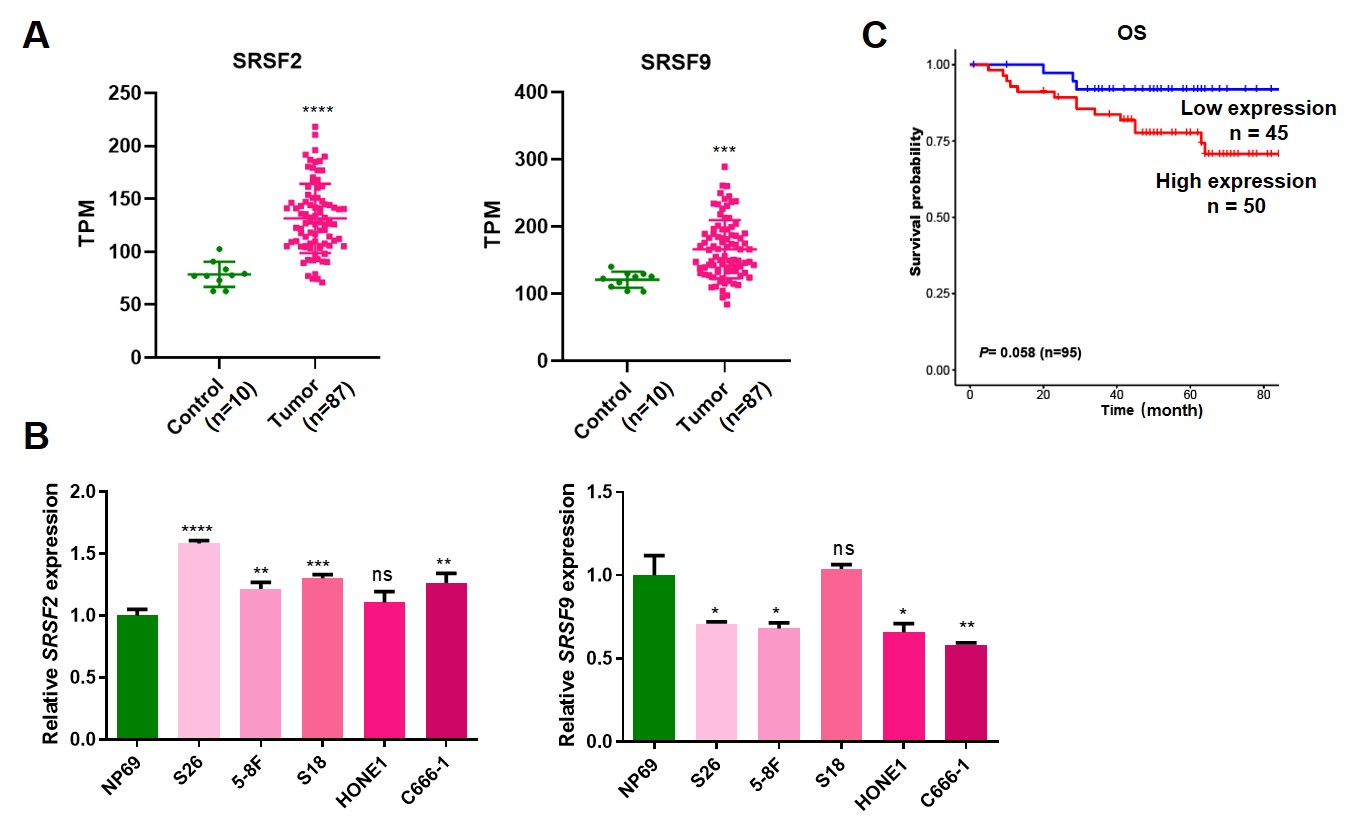


**Figure S1.** **High expression of SRSF3 predicts worse prognosis in patients with NPC. A**) RNA-seq data presented as TPM showed the expressions of SRSF2 and SRSF9 in NPC tumor and control tissues. **B**) qRT-PCR results showed the mRNA expression of *SRSF2* and *SRSF9* in NPC cell lines and NP69. *ACTIN* was used as an internal control. **C**) Kaplan-Meier survival analysis showed the correlation between the protein expression of SRSF3 and overall survival of NPC patients (n=95). **P*<0.05, ***P*<0.01, ****P*<0.001, *****P*<0.0001.

**Figure S2**


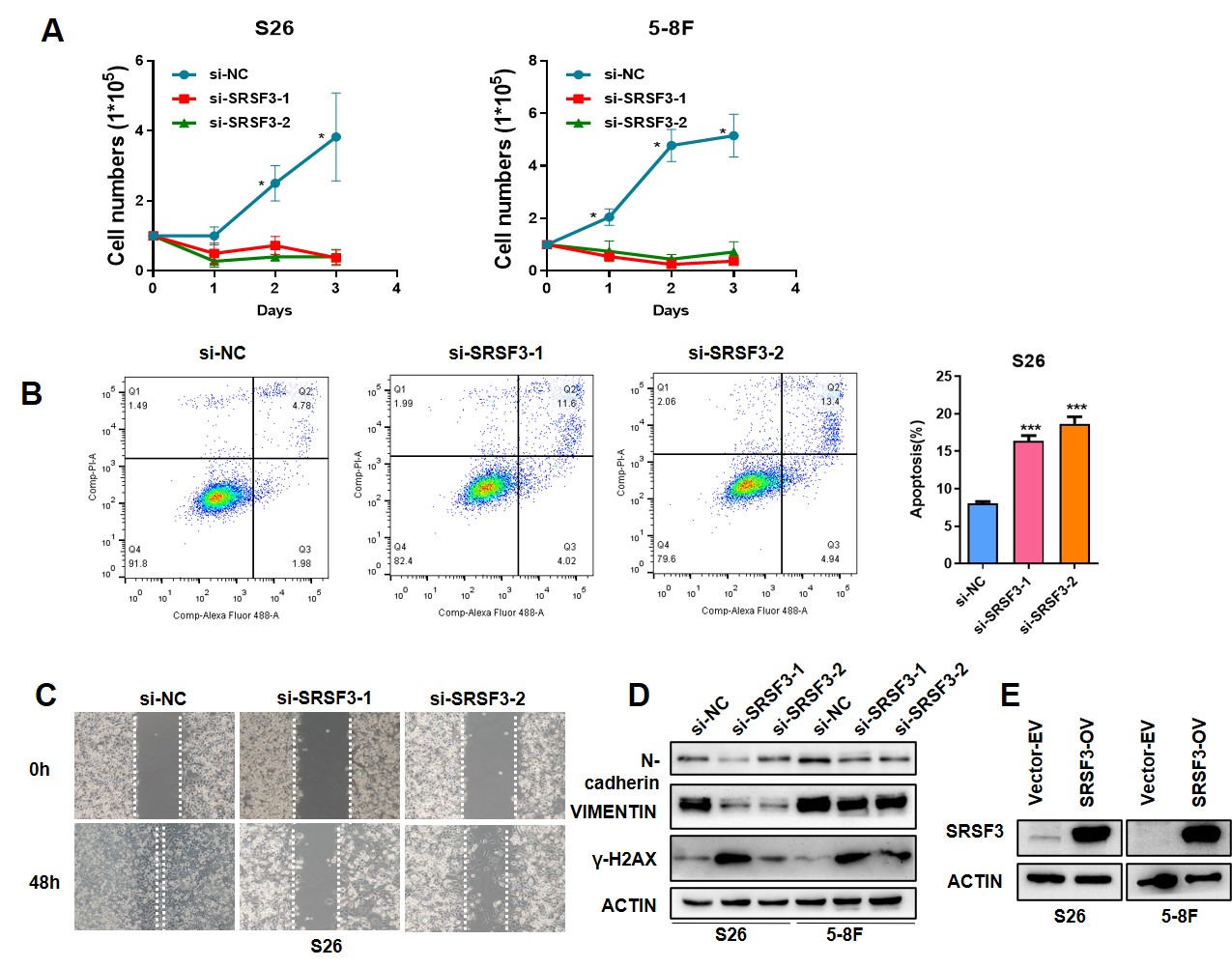


**Figure S2**. **SRSF3 is required for the proliferation and migration of NPC cells. A**) Cell growth curves were measured with S26 and 5-8F cells transfected with siRNAs targeting SRSF3 or control siRNA. **B**) Flow cytometry analysis of Annexin V in S26 cells described in A. Corresponding statistical data were presented at the right. **C**) Wound healing assays were performed with S26 cells described in A. **D**) Western blotting results showed the expression of indicated proteins in cells described in A. **E**) S26 and 5-8F cells were infected with lentivirus expressing SRSF3 or control vectors. Western blotting assay was performed to evaluate the overexpression of SRSF3, with ACTIN treated as control. **P*<0.05, ***P*<0.01, ****P*<0.001.

**Figure S3**


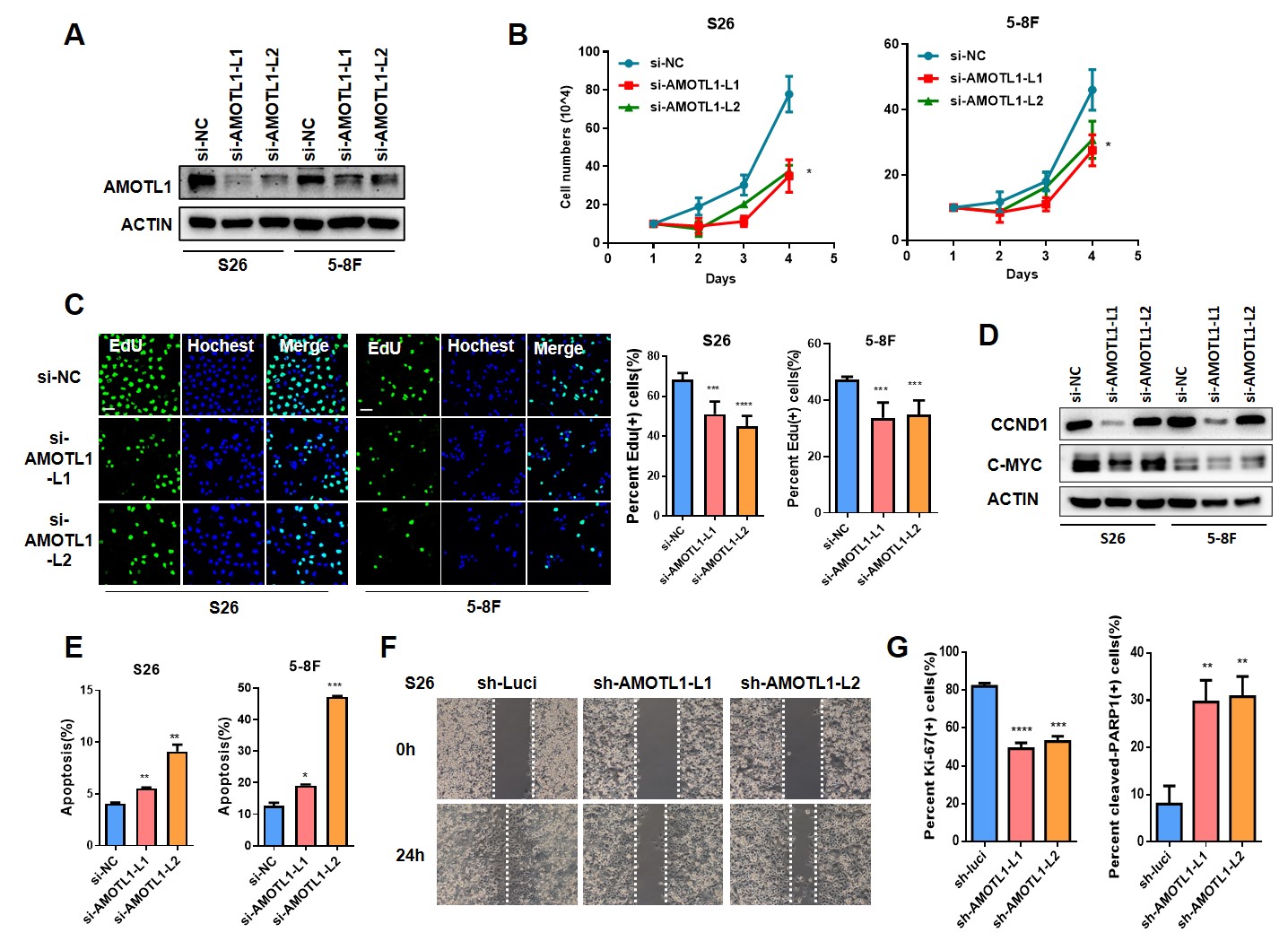


**Figure S3**. **Knockdown of AMOTL1-L decreased the proliferation and migration of NPC cells. A**) S26 and 5-8F cells were transfected with siRNAs specific against exon12 of AMOTL1 or control siRNA. Western blotting resulted showed the knockdown efficiency of AMOTL1-L. ACTIN was used as a loading control. **B**) Cell growth curves were measured with cells described in A. **C**) IF staining of EdU in cells described in A. The statistical analysis was presented at the right. **D**) Western blotting assays were performed to detect the protein expression of CCND1 and c-MYC in cells described in A. ACTIN was used as control. **E**) Flow cytometry analysis of Annexin V demonstrated the apoptosis of cells described in A. **F**) Wound healing assays were carried out with S26 cells infected with lentivirus stably expression shRNA specific targeting AMOTL1 exon12 or control shRNA. **F**) Statistical analysis of cells presented in Figure 5F. Scale bar, 100 μm. **P*<0.05, ***P*<0.01, ****P*<0.001, *****P*<0.0001.

**Figure S4**


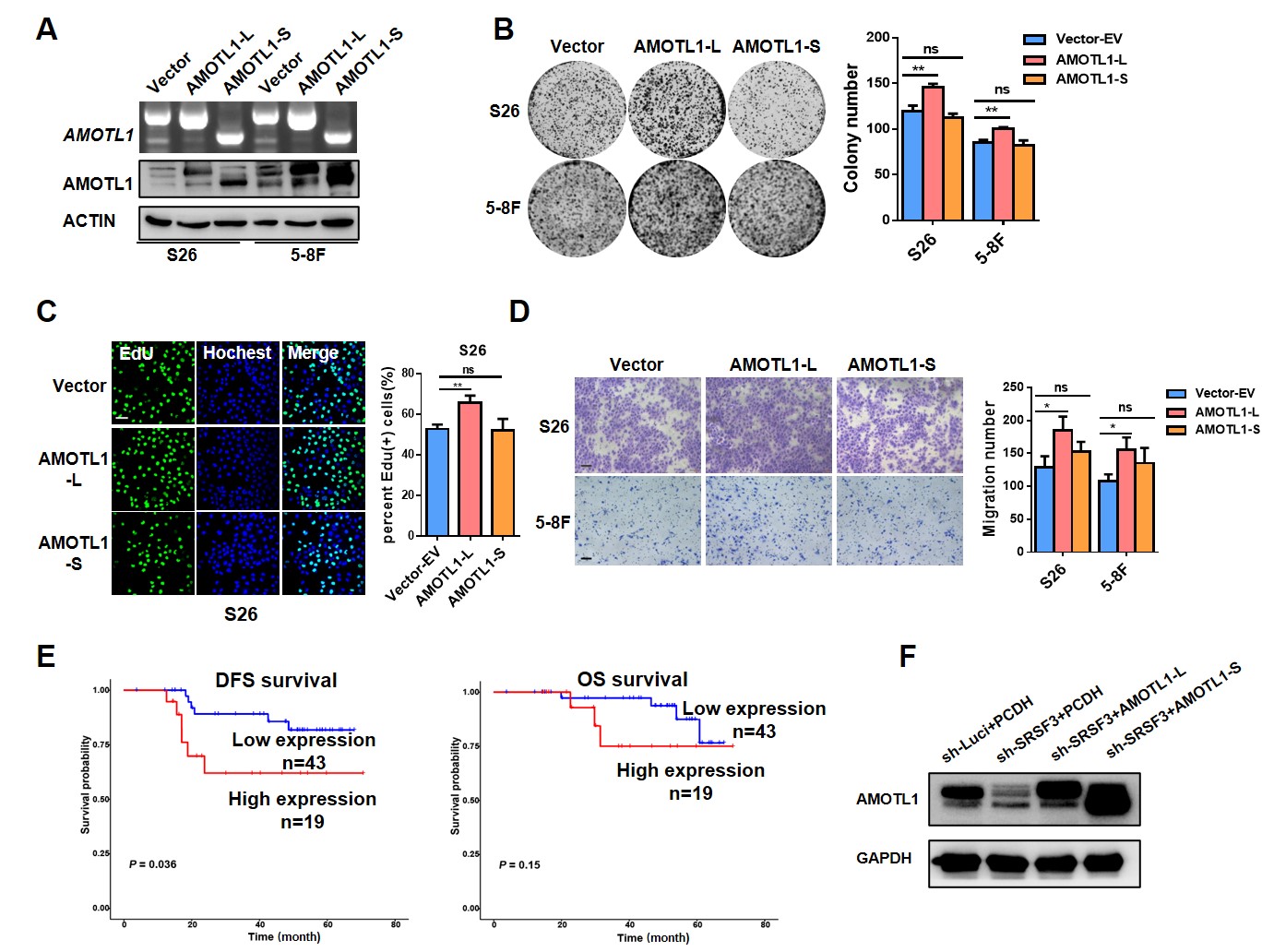


**Figure S4**. **AMOTL1-L mediates the oncogenic function of SRSF3 in NPC. A**) S26 and 5-8F cells were infected with lentivirus stably expressing AMOTL1-L/–S or control vectors. RT-PCR and western blotting results demonstrated the overexpression efficiency. **B**) Colony formation assays were performed with cells described in A. The statistical data were presented at the right. **C**) Representative images of EdU staining were presented with cells described in A and the corresponding statistic was showed at the right. **D**) Transwell assays were performed with cell described in A and the corresponding statistical analysis were demonstrate at the bottom. **E**) Kaplan-Meier survival analysis showed the correlation between the mRNA expression of AMOTL1-L and overall and disease-free survival of NPC patients. **F**) 5-8F cells stably expressing SRSF3 shRNAs or control shRNA were infected with lentivirus expressing AMOTL1-L/-S. Western blotting assay was performed to detect the overexpression efficiency. Scale bar, 100 μm. **P*<0.05, ***P*<0.01, ****P*<0.001.

**Figure S5**


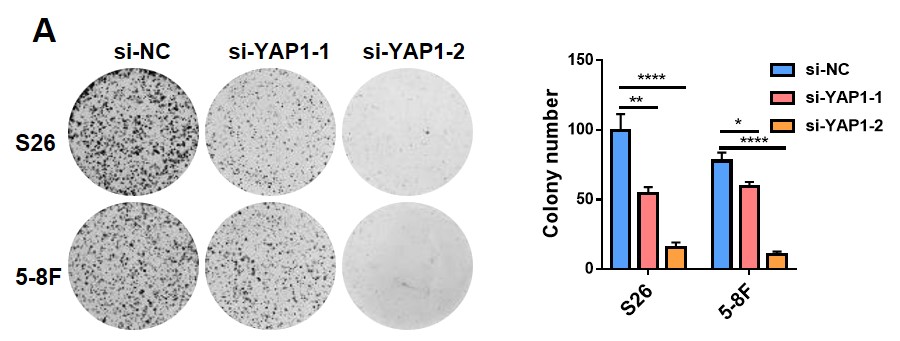


**Figure S5**. **Depletion of YAP1 inhibited the cell growth of NPC cells. A**) S26 and 5-8F cells were transiently transfected with YAP1 siRNAs or control siRNA. Colony formation assays were performed and the corresponding statistic was showed at the right. **P*<0.05, ***P*<0.01, ****P*<0.001, *****P*<0.0001.
