# Supplementary material for: SRSF3/AMOTL1 splicing axis promotes the tumorigenesis of nasopharyngeal carcinoma through regulating the nucleus translocation of YAP1
Source: Cell Death Dis. 2023 Aug 9;14(8):511. doi: 10.1038/s41419-023-06034-1 (PMC10412622; doi:10.1038/s41419-023-06034-1)
Supplement: Supplementary file 2 — Table S1 [file 41419_2023_6034_MOESM2_ESM.docx]

**Table S1. Primer-id sequence (5'-3')**

| SRSF3-Nhe1-F | CTAGCTAGCATGCATCGTGATTCCTGTCCA |
| --- | --- |
| SRSF3-BamH1-R | CGCGGATCCCACCACTTCTCTTGCAAACTG |
| AMOTL1-E1-F | CTACTGGGACACACTCTCGC |
| AMOTL1-E3-R | GGGATGGCAGTTAGATGAGGA |
| TFAP4-E1-F | GCCCACATGTACCCGGAAAA |
| TFAP4-E3-R | GGACAGGCTTCACGATGACA |
| RBM27-E1-F | TCCCTCAGGAATTGAACAACA |
| RBM27-E3-R | ACTCTGGTTACCACCTGCATG |
| MAP4-E1-F | CCAGGCCAGAAGAAGGAAGG |
| MAP4-E3-R | GCAGCAGCTGTTGTGGTTTT |
| THOC5-E1-F | TGCGCTGTTACCTCATCCGAA |
| THOC5-E3-R | CTGCTCGGTGTCAGATCGATT |
| ELOVL1-E1-F | TGGAGGCTGTTGTGAACTTGT |
| ELOVL1-E3-R | TCAGGGCTGTTGGAATAGTCC |
| HNRNPUL1-E1-F | GAACAGCAACATCCCTGGCTC |
| HNRNPUL1-E3-R | TAGTTCCCGTAGTATGGCGGC |
| LRP8-E1-F | CCTGTGAGCTGAGTGTCCAG |
| LRP8-E3-R | CTATGGGCACGATGATCCCG |
| SUPT6H-E1-F | ACAGAACCCTAATGCCACCC |
| SUPT6H-E3-R | CACTGCTCCGCCATTTTTCC |
| SMN2-E1-F | AGTGAGAACTCCAGGTCTCCT |
| SMN2-E3-R | AGCCAGTATGATAGCCACTCA |
| CLIP1-E0-F | TCAGTTCCGGGTTGAAGAAGA |
| CLIP1-E3-R | CAGGCTTATTGGACTCTAGCC |
| GOLGA2-E1-F | GGAATAGCCCTGGTGTTCCT |
| GOLGA2-E3-R | CTGAGATGCCGCCATGCTAG |
| UBAP2L-E1-F | GGAGGACTGCAACAGAGGAG |
| UBAP2L-E3-R | TCTGACCAGCAGTGATTGTCA |
| SMAP1-E0-F | TCCAGAGAACTTTCGAAGACCA |
| SMAP1-E4-R | TCCTAAAAGATCCACAGTGGGC |
| EIF4H-E1-F | ATCGGTCACTTCGTGTGGAC |
| EIF4H-E3-R | CATGTTGGATCCACGGAGGG |
| KLHL2-E1-F | CCCAACAACCGATAAATGGACA |
| KLHL2-E3-R | ACTTGTTGAAGGCTGTTTCTCA |
| ZXDC-E1-F | GCAACAGACTCTTCACCTCCA |
| ZXDC -E3-R | GAACCACTAGCATTGGCAGG |
| FXR1-E1-F | TGCCAGCGAATCTCATCACA |
| FXR1-E3-R | TCCTGGAGTACGCTGTAGCT |
| ASB8-E1-F | CCCTTGCACTGTGCCTGTAT |
| ASB8-E3-R | GTGGAGGGCTGTTCGGTTAT |
| LATS1-E1-F | GTCCTTCGTGTGGGCTACAT |
| LATS1-E3-R | TGCTCTCGTCGAGGATCTTG |
| AMOT1-BstB1-F | GTCTTCGAAGAACTAGCCATGATCGCCTCA |
| AMOT1-Not1-R | GTCGCGGCCGCAAATTCCACAGGGATGGCAGT |
| AMOTL1-Kpn1-mini-F | CGGGGTACCCAGTATTAAAAATCTCCATGCCAAA |
| AMOTL1-Xho1-mini-R | CCGCTCGAGACCATGTGTAATAAAATCCATCGCA |
| SRSF3-RRM1-F | CTCATGCCCGTTAGACGGCTTTGCTTTTGTTGA |
| SRSF3-RRM1-R | TCAACAAAAGCAAAGCCGTCTAACGGGCATGAG |
| SRSF3-RRM2-F | TGCTAGAAACCCACCCGAAAAAAGAAGTAG |
| SRSF3-RRM2-R | CTACTTCTTTTTTCGGGTGGGTTTCTAGCA |
| SRSF3-RS1-F | GAATGGTGAAAAAAGAAGCAGGTCCCTTTCTA |
| SRSF3-RS1-R | TAGAAAGGGACCTGCTTCTTTTTTCACCATTC |
| SRSF3-RS2-F | CTTCTCTCGCAGCCGGTACCCATACGATGTT |
| SRSF3-RS2-R | AACATCGTATGGGTACCGGCTGCGAGAGAAG |
| shluci-F | CCGGTTCCTGGAACAATTGCTTTTACTCGAGTAAAAGCAATTGTTCCAGGAATTTTTG |
| shluci-R | AATTCAAAAATTCCTGGAACAATTGCTTTTACTCGAGTAAAAGCAATTGTTCCAGGAA |
| SRSF3-shRNA-3-F | CCGGAACAGTGACACAAAGGTGTAATTCTCGAGAATTACACCTTTGTGTCACTGTTTTTTTG |
| SRSF3-shRNA-3-R | AATTCAAAAAAACAGTGACACAAAGGTGTAATTCTCGAGAATTACACCTTTGTGTCACTGTT |
| SRSF3-shRNA-4-F | CCGGAACGAGAGCTAGATGGAAGAACACTCGAGTGTTCTTCCATCTAGCTCTCGTTTTTTTG |
| SRSF3-shRNA-4-R | AATTCAAAAAAACGAGAGCTAGATGGAAGAACACTCGAGTGTTCTTCCATCTAGCTCTCGTT |
| SRSF3-siRNA-1 | TCGTGATTCCTGTCCATTG |
| SRSF3-siRNA-2 | TGGCAACAAGACGGAATTG |
| AMOTL1-shRNA-L-F1 | CCGGAACACACAGACTGACAAGAGTCTCGAGACTCTTGTCAGTCTGTGTGTTTTTTTG |
| AMOTL1-shRNA-L-R1 | AATTCAAAAAAACACACAGACTGACAAGAGTCTCGAGACTCTTGTCAGTCTGTGTGTT |
| AMOTL1-shRNA-L-F2 | CCGGAAGCAGCACACAGACTGACAACTCGAGTTGTCAGTCTGTGTGCTGCTTTTTTTG |
| AMOTL1-shRNA-L-R2 | AATTCAAAAAAAGCAGCACACAGACTGACAACTCGAGTTGTCAGTCTGTGTGCTGCTT |
| AMOTL1-shRNA-L-F3 | CCGGAAACAAGAGTGCCGAGCTCTTCTCGAGAAGAGCTCGGCACTCTTGTTTTTTTTG |
| AMOTL1-shRNA-L-R3 | AATTCAAAAAAAACAAGAGTGCCGAGCTCTTCTCGAGAAGAGCTCGGCACTCTTGTTT |
| si-AMOTL1-L1 | CACACAGACTGACAAGAGT |
| si-AMOTL1-L2 | GCAGCACACAGACTGACAA |
| AMOTL1-L-F | ACTCTCGCCAGACCTCTCTT |
| AMOTL1-L-R | TTTCTCTGCGGTCCCTTTGG |
| AMOTL1-S-F | TGATTAAGGTCCTGCAGCAGC |
| AMOTL1-S-R | GGCCAGGAGAGTTCTCTATGC |
| AMOTL1-mini-F | GCTTGGTACCCAGTATTAA |
| AMOTL1-mini-R | TAGACTCGAGACCATGTGTA |
| YAP1-F | CAGCATGTTCGAGCTCATTCC |
| YAP1-R | TCTCTGACCAGAAGATGTCTTTG |
| β-actin-F | CCCACACTGTGCCCATCTAC |
| β-actin-R | GGAACCGCTCATTGCCAATG |
| SRSF9-F | TGGTTATGATTATGGCCAGTGT |
| SRSF9-R | GCATAACAGACATCCCCAGCT |
| SRSF2-F | CTACAGCCGCTCGAAGTCTC |
| SRSF2-R | TTGGATTCCCTCTTGGACAC |
| SRSF3-F | AACAAGACGGAATTGGAACG |
| SRSF3-R | TGGGCCACGATTTCTACTTC |
| AMOTL1-RIP-E1-F | TATTAAAAATCTCCATGCCAAA |
| AMOTL1- RIP-E1-R | CTATGCTCCCCTTCCAGGTC |
| AMOTL1- RIP-E2-F | GATTGCTGCTGGGGAAGGAGC |
| AMOTL1- RIP-E2-R | CCAGTTTCTCTGCGGTCCCT |
| AMOTL1- RIP-E3-F | AGAACTCTCCTGGCCATGGG |
| AMOTL1- RIP-E3-R | TCTGAAATCTTTATCAGTTCA |
| SRSF3-HA-F | GGTCAAATGAAAGGAAATACCCATACGATGTTCCAGATTACGCTTAGAAGACAGTTTGC |
| SRSF3-HA-R | GCAAACTGTCTTCTAAGCGTAATCTGGAACATCGTATGGGTATTTCCTTTCATTTGACC |
| AMOTL-HA-F | TGGAAGTCCTCATCTACCCATACGATGTTCCAGATTACGCTTAACTGCCATCCCTGT |
| AMOTL-HA-F | ACAGGGATGGCAGTTAAGCGTAATCTGGAACATCGTATGGGTAGATGAGGACTTCCA |

**Table S1**. List of Specific primers.
