# Supplementary figures and images for: SRSF3/AMOTL1 splicing axis promotes the tumorigenesis of nasopharyngeal carcinoma through regulating the nucleus translocation of YAP1
Source: Cell Death Dis. 2023 Aug 9;14(8):511. doi: 10.1038/s41419-023-06034-1 (PMC10412622; doi:10.1038/s41419-023-06034-1)

Figure 1

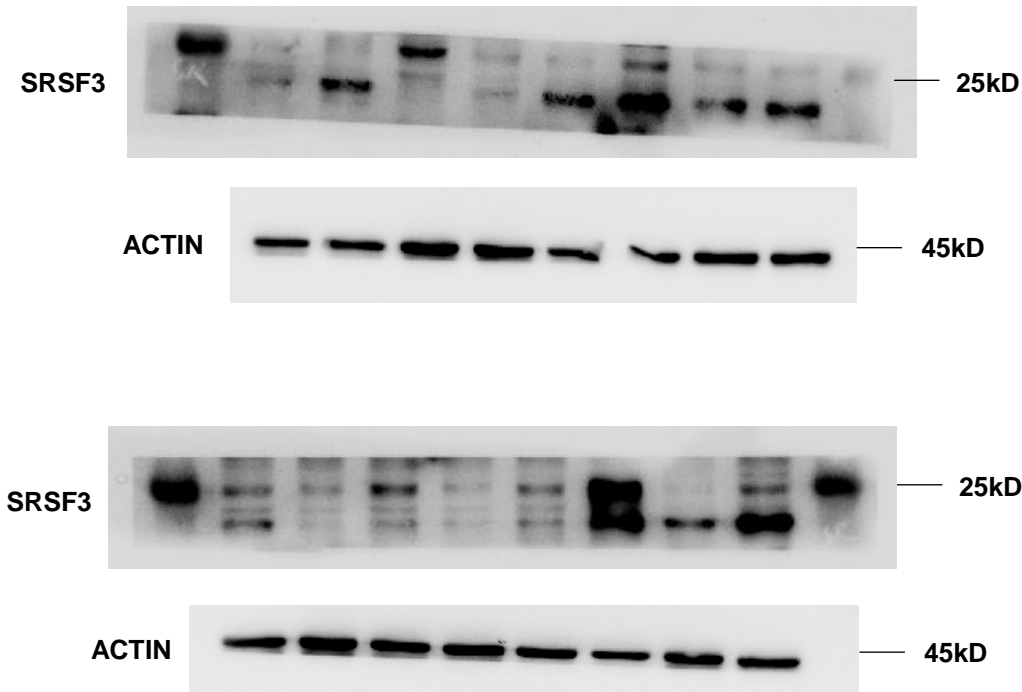

Figure 2

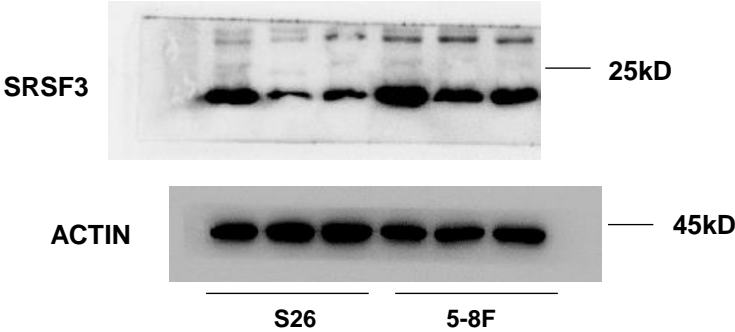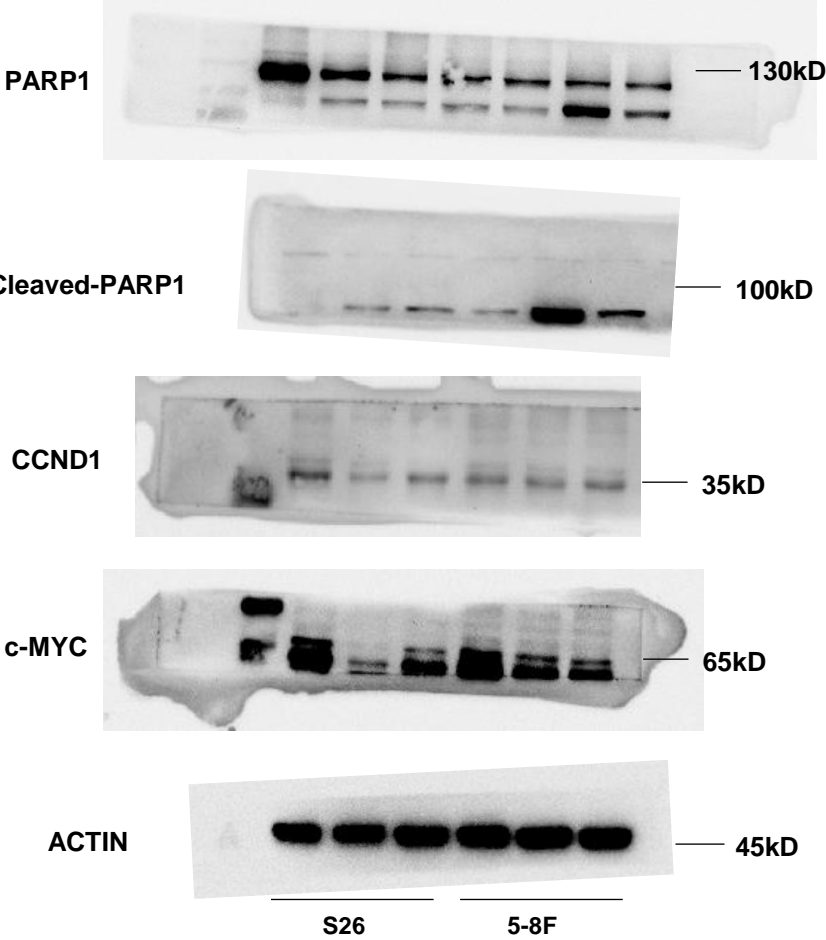

Figure 4

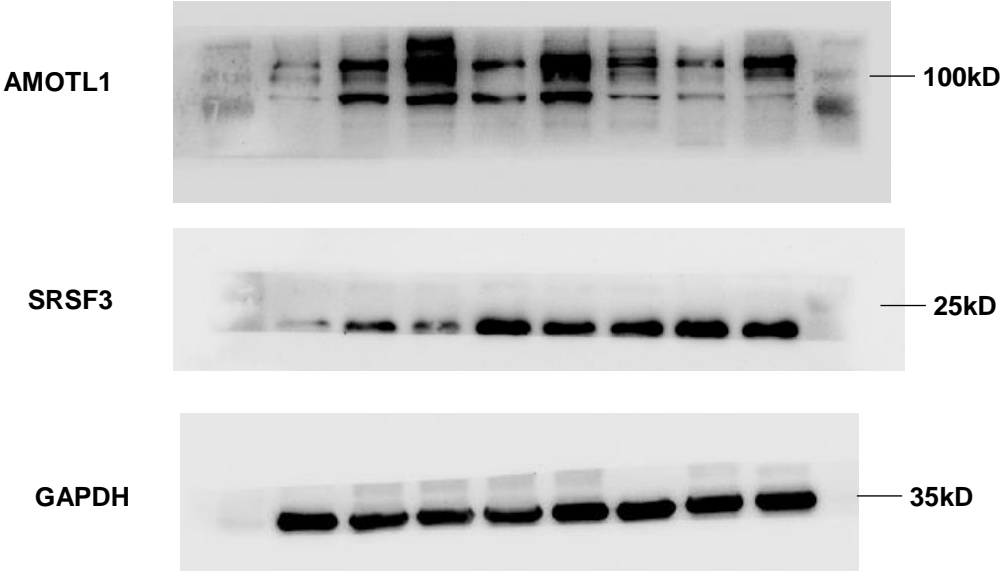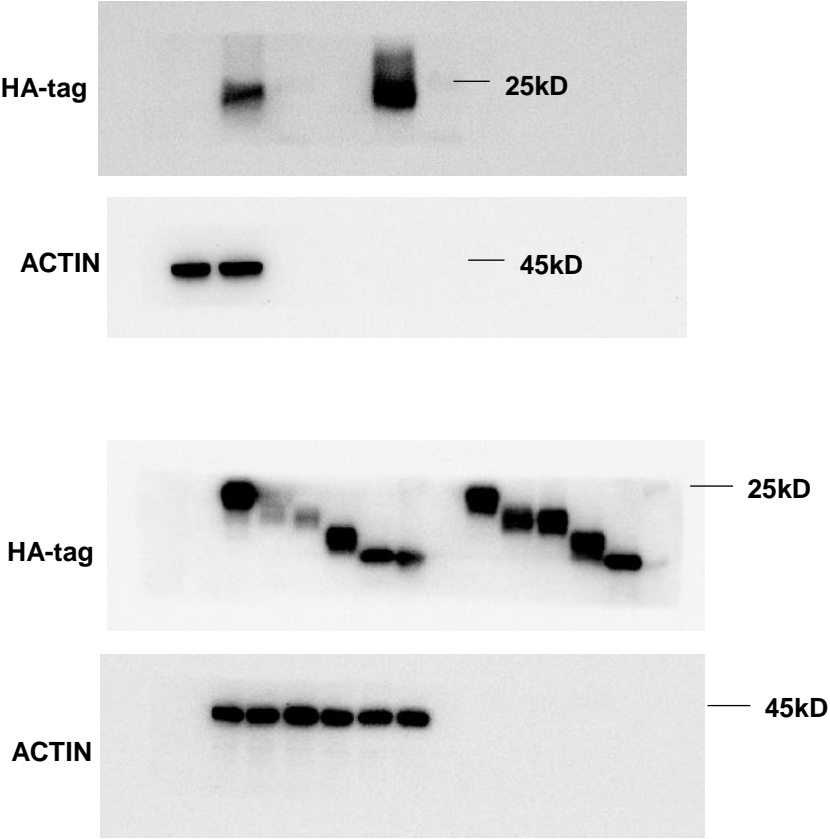

Figure 6

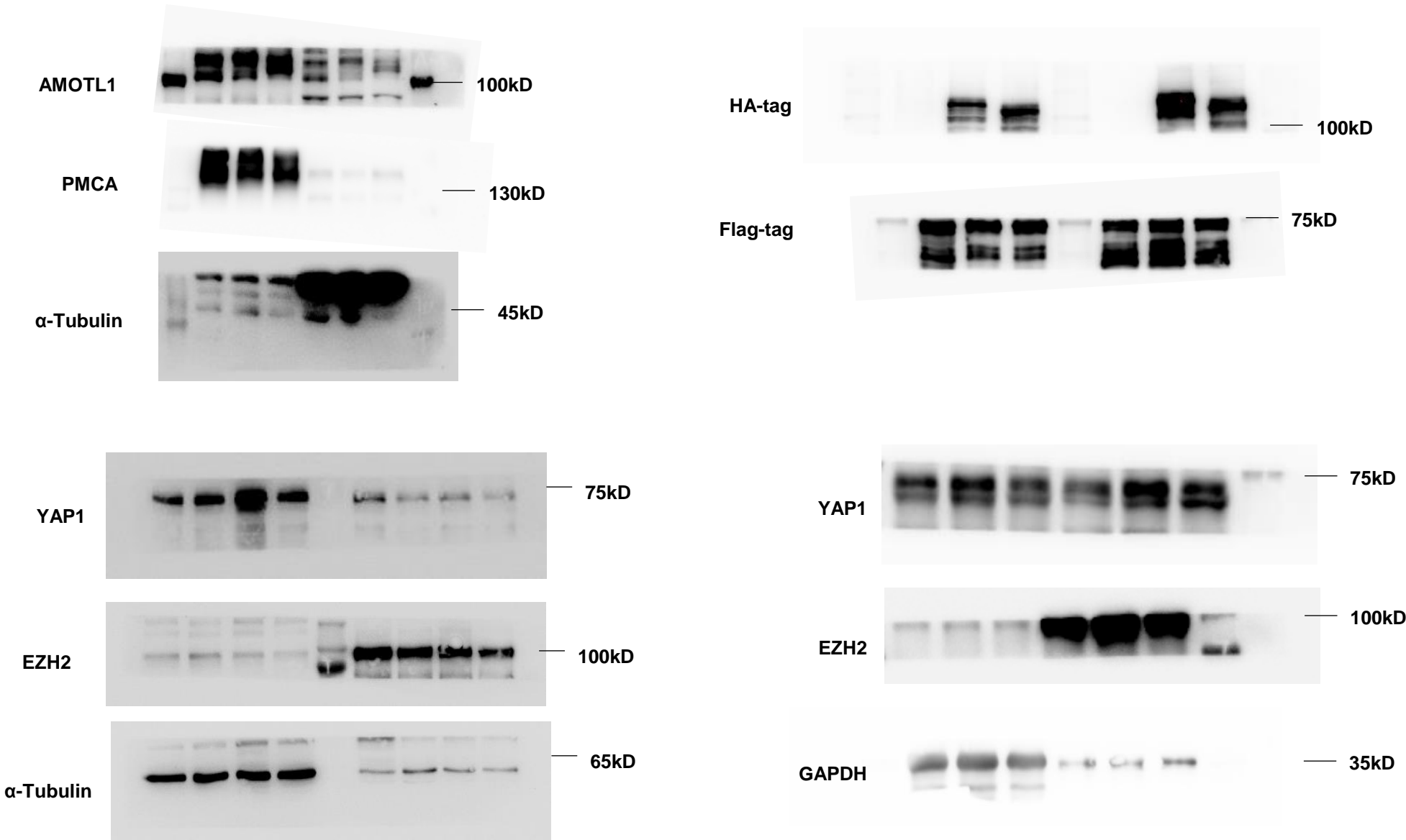

**Figure 6-2**

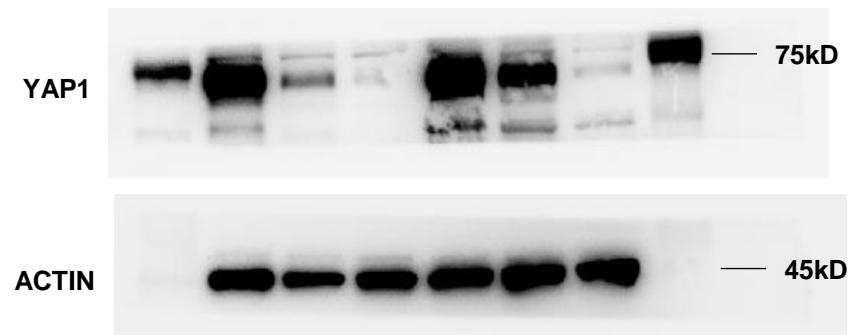

Figure S2

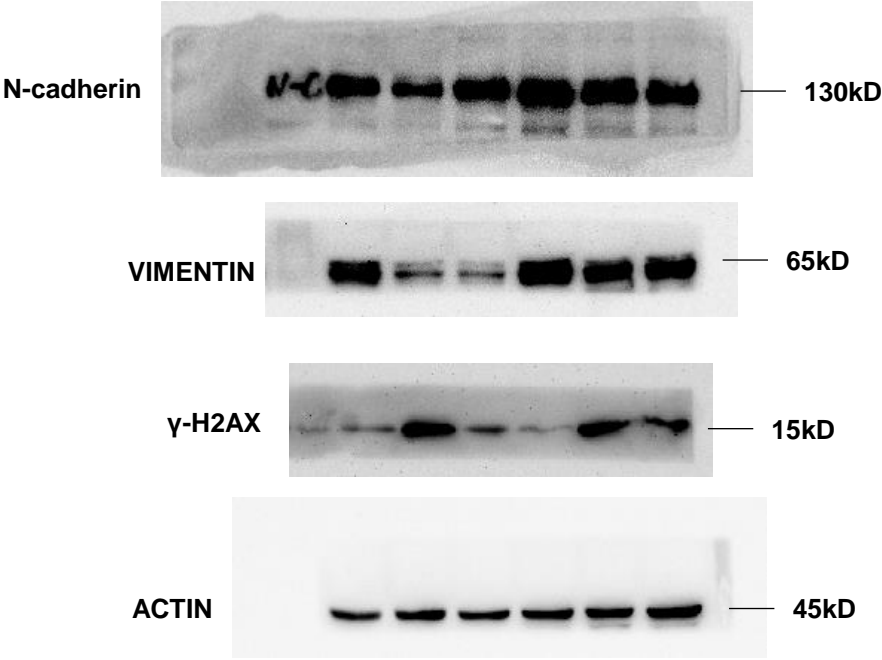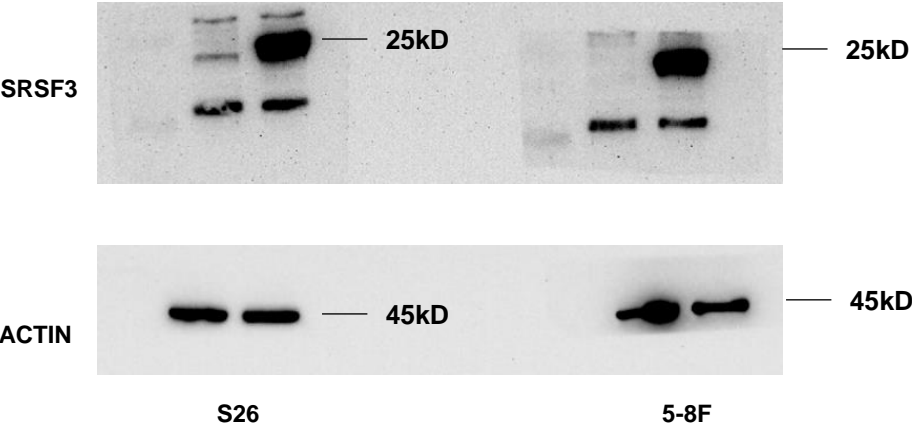

Figure S3

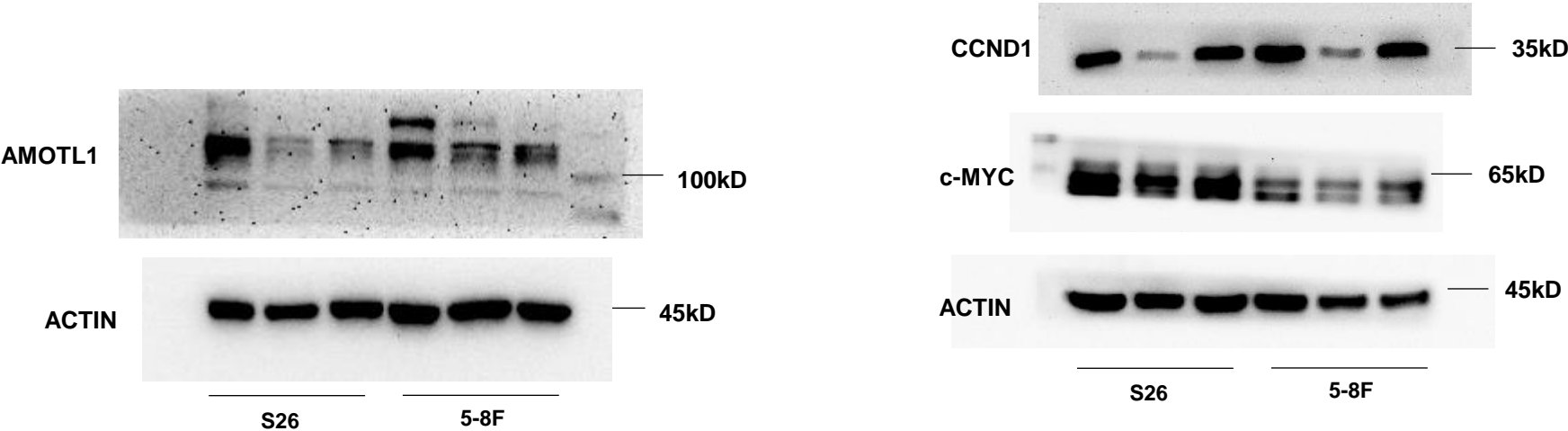

**Figure S4**

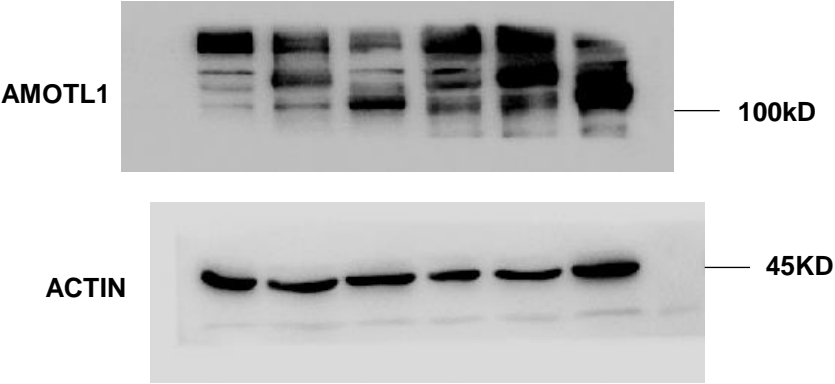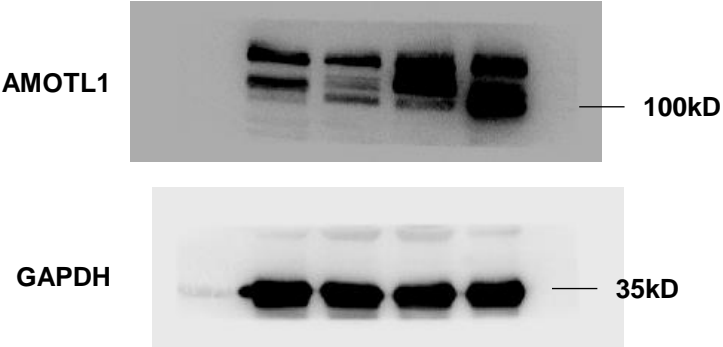

Supplement: Supplementary file 3 — Original Data File [file 41419_2023_6034_MOESM3_ESM.pdf]
