# Supplementary material for: SRSF3/AMOTL1 splicing axis promotes the tumorigenesis of nasopharyngeal carcinoma through regulating the nucleus translocation of YAP1
Source: Cell Death Dis. 2023 Aug 9;14(8):511. doi: 10.1038/s41419-023-06034-1 (PMC10412622; doi:10.1038/s41419-023-06034-1)
Supplement: Supplementary file 4 — Confirmation of changes in Authorship [file 41419_2023_6034_MOESM4_ESM.pdf]

Re:Confirmation of changes in Authorship

发件人：中山医学院徐晓辰<xxc940514@163.com>
时　间：2023年7月28日(星期五) 凌晨0:26
收件人：罗春玲<luochl@sysucc.org.cn>
抄　送：beijx<beijx@sysucc.org.cn>; 蒋家馨<jiangjx@sysucc.org.cn>; 何帅<heshuai@sysucc.org.cn>; 刘洋<liuyang3@sysucc.org.cn>; 李艺祺<liyq2@sysucc.org.cn>; weipp<weipp@sysucc.org.cn>; 周雅青<zhouyq@sysucc.org.cn>; sjian<sjian@mail.sysu.edu.cn>

Yes, I agree to the author list being changed

在 2023-07-27 23:10:47，"罗春玲" <luochl@sysucc.org.cn> 写道：

> Dear Co-authors,
>
> As mentioned before, our manuscript (**CDDIS-23-1676**) has been accepted in principle by *Cell Death & Disease*. We are preparing the very final revision. We changed the author list by adding Yi-Qi Li who contribution in the revisions. Please check below.
>
> The current author list is — Xiao-Chen Xu#, Jia-Xin Jiang#, Ya-Qing Zhou, Shuai He, Yang Liu, Yi-Qi Li, Pan-Pan Wei, Jin-Xin Bei, Jian Sun*, Chun-Ling Luo*.
>
> #contributed equally,*corresponding authors.
>
> The previous author list was — Xiao-Chen Xu#, Jia-Xin Jiang#, Ya-Qing Zhou, Shuai He, Yang Liu, Pan-Pan Wei, Jin-Xin Bei, Jian Sun*, Chun-Ling Luo*.
>
> If you agree to the authorship arrangement, please confirm the change of the authorship by replying this email with "**Yes, I agree to the author list being changed**".
>
> Thank you very much.
>
> Chunling

回复:Confirmation of changes in Authorship

发件人：蒋家馨<jiangjx@sysucc.org.cn>
时　间：2023年7月27日(星期四) 晚上11:27
收件人：罗春玲<luochl@sysucc.org.cn>

Yes, I agree to the author list being changed.

--------------原始邮件--------------
发件人："罗春玲 "<luochl@sysucc.org.cn>;
发送时间：2023年7月27日(星期四) 晚上11:10
收件人："贝锦新" <beijx@sysucc.org.cn>;"xxc940514" <xxc940514@163.com>;"蒋家馨" <jiangjx@sysucc.org.cn>;"何帅" <heshuai@sysucc.org.cn>;"刘洋" <liuyang3@sysucc.org.cn>;"李艺祺" <liyq2@sysucc.org.cn>;"weipp@sysucc.org.cn" <weipp@sysucc.org.cn>;"周雅青" <zhouyq@sysucc.org.cn>;"sjian" <sjian@mail.sysu.edu.cn>;
主题：Confirmation of changes in Authorship

Dear Co-authors,

As mentioned before, our manuscript (**CDDIS-23-1676**) has been accepted in principle by *Cell Death & Disease*. We are preparing the very final revision. We changed the author list by adding Yi-Qi Li who contribution in the revisions. Please check below.

The current author list is — Xiao-Chen Xu[#], Jia-Xin Jiang[#], Ya-Qing Zhou, Shuai He, Yang Liu, Yi-Qi Li, Pan-Pan Wei, Jin-Xin Bei, Jian Sun[*], Chun-Ling Luo[*].

#contributed equally,*corresponding authors.

The previous author list was — Xiao-Chen Xu[#], Jia-Xin Jiang[#], Ya-Qing Zhou, Shuai He, Yang Liu, Pan-Pan Wei, Jin-Xin Bei, Jian Sun[*], Chun-Ling Luo[*].

If you agree to the authorship arrangement, please confirm the change of the authorship by replying this email with "**Yes, I agree to the author list being changed**".

Thank you very much.

Chunling

回复:Confirmation of changes in Authorship

发件人：周雅青<zhouyq@sysucc.org.cn>
时　间：2023年7月27日(星期四) 晚上11:14
收件人：罗春玲<luochl@sysucc.org.cn>

Yes, I agree to the author list being changed.
--------------原始邮件--------------
发件人："罗春玲 "<luochl@sysucc.org.cn>;
发送时间：2023年7月27日(星期四) 晚上11:10
收件人："beijx" <beijx@sysucc.org.cn>;"xxc940514" <xxc940514@163.com>;"蒋家馨" <jiangjx@sysucc.org.cn>;"何帅" <heshuai@sysucc.org.cn>;"刘洋" <liuyang3@sysucc.org.cn>;"李艺祺" <liyq2@sysucc.org.cn>;"weipp" <weipp@sysucc.org.cn>;"周雅青" <zhouyq@sysucc.org.cn>;"sjian" <sjian@mail.sysu.edu.cn>;
主题：Confirmation of changes in Authorship

Dear Co-authors,

As mentioned before, our manuscript (**CDDIS-23-1676**) has been accepted in principle by *Cell Death & Disease*. We are preparing the very final revision. We changed the author list by adding Yi-Qi Li who contribution in the revisions. Please check below.

The current author list is — Xiao-Chen Xu#, Jia-Xin Jiang#, Ya-Qing Zhou, Shuai He, Yang Liu, Yi-Qi Li, Pan-Pan Wei, Jin-Xin Bei, Jian Sun*, Chun-Ling Luo*.

#contributed equally,*corresponding authors.

The previous author list was — Xiao-Chen Xu#, Jia-Xin Jiang#, Ya-Qing Zhou, Shuai He, Yang Liu, Pan-Pan Wei, Jin-Xin Bei, Jian Sun*, Chun-Ling Luo*.

If you agree to the authorship arrangement, please confirm the change of the authorship by replying this email with "**Yes, I agree to the author list being changed**".

Thank you very much.

Chunling

回复:Confirmation of changes in Authorship

发件人：何帅<heshuai@sysucc.org.cn>
时　间：2023年7月27日(星期四) 晚上11:14
收件人：罗春玲<luochl@sysucc.org.cn>

Yes, I agree to the author list being changed

该邮件从移动设备发送

--------------原始邮件--------------
发件人："罗春玲 "<luochl@sysucc.org.cn>;
发送时间：2023年7月27日(星期四) 晚上11:10
收件人："贝锦新" <beijx@sysucc.org.cn>;"中山医学院徐晓辰" <xxc940514@163.com>;"蒋家馨" <jiangjx@sysucc.org.cn>;"何帅" <heshuai@sysucc.org.cn>;"刘洋" <liuyang3@sysucc.org.cn>;"李艺祺" <liyq2@sysucc.org.cn>;"魏盼盼" <weipp@sysucc.org.cn>;"周雅青" <zhouyq@sysucc.org.cn>;"sjian" <sjian@mail.sysu.edu.cn>;
主题：Confirmation of changes in Authorship

Dear Co-authors,

As mentioned before, our manuscript (**CDDIS-23-1676**) has been accepted in principle by *Cell Death & Disease*. We are preparing the very final revision. We changed the author list by adding Yi-Qi Li who contribution in the revisions. Please check below.

The current author list is — Xiao-Chen Xu[#], Jia-Xin Jiang[#], Ya-Qing Zhou, Shuai He, Yang Liu, Yi-Qi Li, Pan-Pan Wei, Jin-Xin Bei, Jian Sun[*], Chun-Ling Luo[*].

#contributed equally,*corresponding authors.

The previous author list was — Xiao-Chen Xu[#], Jia-Xin Jiang[#], Ya-Qing Zhou, Shuai He, Yang Liu, Pan-Pan Wei, Jin-Xin Bei, Jian Sun[*], Chun-Ling Luo[*].

If you agree to the authorship arrangement, please confirm the change of the authorship by replying this email with "**Yes, I agree to the author list being changed**".

Thank you very much.

Chunling

回复:Confirmation of changes in Authorship

发件人：刘洋（2018级直博生）<liuyang3@sysucc.org.cn>
时　间：2023年7月27日(星期四) 晚上11:16
收件人：罗春玲<luochl@sysucc.org.cn>

Yes, I agree to the author list being changed！

--------------原始邮件--------------
发件人："罗春玲 "<luochl@sysucc.org.cn>;
发送时间：2023年7月27日(星期四) 晚上11:10
收件人："beijx" <beijx@sysucc.org.cn>;"中山医学院徐晓辰" <xxc940514@163.com>;"蒋家馨" <jiangjx@sysucc.org.cn>;"何帅" <heshuai@sysucc.org.cn>;"刘洋" <liuyang3@sysucc.org.cn>;"李艺祺" <liyq2@sysucc.org.cn>;"weipp" <weipp@sysucc.org.cn>;"周雅青" <zhouyq@sysucc.org.cn>;"sjian" <sjian@mail.sysu.edu.cn>;
主题：Confirmation of changes in Authorship

Dear Co-authors,

As mentioned before, our manuscript (**CDDIS-23-1676**) has been accepted in principle by *Cell Death & Disease*. We are preparing the very final revision. We changed the author list by adding Yi-Qi Li who contribution in the revisions. Please check below.

The current author list is — Xiao-Chen Xu#, Jia-Xin Jiang#, Ya-Qing Zhou, Shuai He, Yang Liu, Yi-Qi Li, Pan-Pan Wei, Jin-Xin Bei, Jian Sun*, Chun-Ling Luo*.

#contributed equally,*corresponding authors.

The previous author list was — Xiao-Chen Xu#, Jia-Xin Jiang#, Ya-Qing Zhou, Shuai He, Yang Liu, Pan-Pan Wei, Jin-Xin Bei, Jian Sun*, Chun-Ling Luo*.

If you agree to the authorship arrangement, please confirm the change of the authorship by replying this email with "**Yes, I agree to the author list being changed**".

Thank you very much.

Chunling

Re: Confirmation of changes in Authorship

发件人：李艺祺<liyq2@sysucc.org.cn>
时　间：2023年7月27日(星期四) 晚上11:19
收件人：罗春玲<luochl@sysucc.org.cn>

Yes, I agree to the author list being changed.

Regards,
Yiqi

From: 罗春玲
Date: 2023-07-27 23:10
To: beijx; 中山医学院徐晓辰; 蒋家馨; 何帅; 刘洋; 李艺祺; weipp; 周雅青; sjian
Subject: Confirmation of changes in Authorship

Dear Co-authors,

As mentioned before, our manuscript (**CDDIS-23-1676**) has been accepted in principle by *Cell Death & Disease*. We are preparing the very final revision. We changed the author list by adding Yi-Qi Li who contribution in the revisions. Please check below.
The current author list is — Xiao-Chen Xu#, Jia-Xin Jiang#, Ya-Qing Zhou, Shuai He, Yang Liu, Yi-Qi Li, Pan-Pan Wei, Jin-Xin Bei, Jian Sun*, Chun-Ling Luo*.
#contributed equally,*corresponding authors.
The previous author list was — Xiao-Chen Xu#, Jia-Xin Jiang#, Ya-Qing Zhou, Shuai He, Yang Liu, Pan-Pan Wei, Jin-Xin Bei, Jian Sun*, Chun-Ling Luo*.
If you agree to the authorship arrangement, please confirm the change of the authorship by replying this email with "**Yes, I agree to the author list being changed**".
Thank you very much.
Chunling

Re: Confirmation of changes in Authorship

发件人：weipp<weipp@sysucc.org.cn>
时　间：2023年7月27日(星期四) 晚上11:26
收件人：罗春玲<luochl@sysucc.org.cn>

Yes, I agree to the author list being changed

Wei Panpan

------------------ Original ------------------

From: 罗春玲 <luochl@sysucc.org.cn>
Date: Thu,Jul 27,2023 11:10 PM
To: beijx <beijx@sysucc.org.cn>, 中山医学院徐晓辰 <xxc940514@163.com>, 蒋家謦 <jiangjx@sysucc.org.cn>, 何帅 <heshuai@sysucc.org.cn>, 刘洋 <liuyang3@sysucc.org.cn>, 李艺祺 <liyq2@sysucc.org.cn>, weipp <weipp@sysucc.org.cn>, 周雅青 <zhouyq@sysucc.org.cn>, sjian <sjian@mail.sysu.edu.cn>
Subject: Re: Confirmation of changes in Authorship

Dear Co-authors,

As mentioned before, our manuscript (**CDDIS-23-1676**) has been accepted in principle by *Cell Death & Disease*. We are preparing the very final revision. We changed the author list by adding Yi-Qi Li who contribution in the revisions. Please check below.

The current author list is — Xiao-Chen Xu#, Jia-Xin Jiang#, Ya-Qing Zhou, Shuai He, Yang Liu, Yi-Qi Li, Pan-Pan Wei, Jin-Xin Bei, Jian Sun*, Chun-Ling Luo*.

#contributed equally,*corresponding authors.

The previous author list was — Xiao-Chen Xu#, Jia-Xin Jiang#, Ya-Qing Zhou, Shuai He, Yang Liu, Pan-Pan Wei, Jin-Xin Bei, Jian Sun*, Chun-Ling Luo*.

If you agree to the authorship arrangement, please confirm the change of the authorship by replying this email with "**Yes, I agree to the author list being changed**".

Thank you very much.

Chunling

Re: Confirmation of changes in Authorship

发件人：BEI\x26nbsp;JINXIN<beijx@sysucc.org.cn>
时　间：2023年7月28日(星期五) 上午8:58
收件人：罗春玲<luochl@sysucc.org.cn>
抄　送：中山医学院徐晓辰<xxc940514@163.com>; 蒋家馨<jiangjx@sysucc.org.cn>; 何帅<heshuai@sysucc.org.cn>; 刘洋<liuyang3@sysucc.org.cn>; 李艺祺<liyq2@sysucc.org.cn>; weipp<weipp@sysucc.org.cn>; 周雅青<zhouyq@sysucc.org.cn>; sjian<sjian@mail.sysu.edu.cn>

Yes, I agree to the author list being changed

Best Regards,

Jinxin

:)

Jin-Xin BEI, Ph.D.
Principal Investigator
State Key Laboratory of Oncology in South China
Sun Yat-sen University Cancer Center
Guangzhou 510060, China
E-mail: beijx@sysucc.org.cn
Tel: +86 20 8734 3189 or +86 20 3933 6779

This email and its attachments may contain confidential information intended for a specific individual and purpose. If you are not the intended recipient, you should delete this email and notify the sender immediately. Any use, dissemination, distribution, or copying of this email or its attachments by persons other than the intended recipient(s), is strictly prohibited.
本邮件及其附件含有发送给特定个人和用于特定目的的保密信息。如果您不是预期的收件人，请立即删除本邮件并通知发件人。严禁任何非预期的收件人使用、传播、分发或复制本邮件或其附件。

On Jul 27, 2023, at 23:10, 罗春玲 <luochl@sysucc.org.cn> wrote:

Dear Co-authors,

As mentioned before, our manuscript (**CDDIS-23-1676**) has been accepted in principle by *Cell Death & Disease*. We are preparing the very final revision. We changed the author list by adding Yi-Qi Li who contribution in the revisions. Please check below.

The current author list is — Xiao-Chen Xu#, Jia-Xin Jiang#, Ya-Qing Zhou, Shuai He, Yang Liu, Yi-Qi Li, Pan-Pan Wei, Jin-Xin Bei, Jian Sun*, Chun-Ling Luo*.

#contributed equally,*corresponding authors.

The previous author list was — Xiao-Chen Xu#, Jia-Xin Jiang#, Ya-Qing Zhou, Shuai He, Yang Liu, Pan-Pan Wei, Jin-Xin Bei, Jian Sun*, Chun-Ling Luo*.

If you agree to the authorship arrangement, please confirm the change of the authorship by replying this email with "**Yes, I agree to the author list being changed**".

Thank you very much.

Chunling

Re:Confirmation of changes in Authorship

发件人：孙健<sjian@mail.sysu.edu.cn>
时　间：2023年7月28日(星期五) 上午10:06
收件人：罗春玲<luochl@sysucc.org.cn>

Yes, I agree to the author list being changed
SunJian

在 2023-07-27 23:10:47，"罗春玲" <luochl@sysucc.org.cn> 写道：

>Dear Co-authors,
>
>As mentioned before, our manuscript (CDDIS-23-1676) has been accepted in principle by Cell Death & Disease. We are preparing the very final revision. We changed the author list by adding Yi-Qi Li who contribution in the revisions. Please check below.
>
>The current author list is — Xiao-Chen Xu#, Jia-Xin Jiang#, Ya-Qing Zhou, Shuai He, Yang Liu, Yi-Qi Li, Pan-Pan Wei, Jin-Xin Bei, Jian Sun*, Chun-Ling Luo*.
>
>#contributed equally,*corresponding authors.
>
>The previous author list was — Xiao-Chen Xu#, Jia-Xin Jiang#, Ya-Qing Zhou, Shuai He, Yang Liu, Pan-Pan Wei, Jin-Xin Bei, Jian Sun*, Chun-Ling Luo*.
>
>If you agree to the authorship arrangement, please confirm the change of the authorship by replying this email with “Yes, I agree to the author list being changed”.
>
>Thank you very much.
>
>Chunling

本邮件及其附件含有发送给特定个人和用于特定目的的信息。如果您不是预期的收件人，请立即删除本邮件并通知发件人。严禁任何非预期的收件人使用、传播、分发或复制本邮件或其附件。

This email and its attachments may contain confidential information intended for a specific individual and purpose. If you are not the intended recipient, you should delete this email and notify the sender immediately. Any use, dissemination, distribution, or copying of this email or its attachments by persons other than the intended recipient(s), is strictly prohibited.
